# Divergent demographic responses to the Kikai-Akahoya volcanic catastrophe in mid-Holocene Japan

**DOI:** 10.1126/sciadv.aeh7197

**Published:** 2026-10-14

**Authors:** Enrico R. Crema, Mitsuhiro Kuwahata, Junzo Uchiyama, Ari Junno, Felix Riede, Peter D. Jordan

**Affiliations:** ^1^ Department of Archaeology, University of Cambridge, Cambridge, UK.; ^2^ Advanced Asian Archaeological Research Centre, Kyushu University, Fukuoka, Japan.; ^3^ Department of Archaeology and Ancient History, Lund University, Helgonavägen 3, 223 62 Lund, Sweden.; ^4^ Sainsbury Institute for the Study of Japanese Arts and Cultures, University of East Anglia, Norwich, UK.; ^5^ Institute for the Study of Ancient Civilizations and Cultural Resources, Kanazawa University, Kanazawa, Japan.; ^6^ Archaeology, Research Unit of History, Culture, and Communication, University of Oulu, P.O. Box 8000, 90014 Oulu, Finland.; ^7^ Department of Archaeology and Heritage Studies, Aarhus University, Aarhus, Denmark.; ^8^ Global Station for Indigenous Studies and Cultural Diversity (GSI), GI-CoRE, Hokkaido University, Sapporo, Japan.

## Abstract

We examine human demographic responses to one of the Holocene’s largest volcanic eruptions, which devastated East Asia 7300 years ago. Archaeologists have intensively debated the impacts of massive environmental shocks, highlighting either societal collapse or cultural continuity, although few studies situate such catastrophic events within longer-term demographic trajectories. We address this gap in knowledge by using statistical models to infer the demographic and environmental impacts of the Kikai-Akahoya (K-Ah) eruption on Jomon foraging communities occupying the Japanese archipelago. Traditionally framed as an apocalyptic “forcing” mechanism that changed the direction of Japanese prehistory, our results identify a more complex and contingent suite of demographic outcomes. Although we do find evidence of major population collapse in immediately adjacent regions to the volcano, some proximal populations actually increased after the catastrophe, whereas some distal communities were affected by environmental consequences that have hitherto been overlooked. Demographic perspectives on ancient disasters are useful in generating a deeper understanding of the contingent ways in which cultures and societies cope with sudden shocks and catastrophes.

## INTRODUCTION

This contribution examines the complex and often unexpected ways in which massive environmental shocks and disasters can affect human demography. We focus on one of the Holocene’s largest ever volcanic disasters (*1*), the Kikai-Akahoya (K-Ah) eruption, which devastated the Japanese archipelago 7300 years ago (Fig. 1). Our main goal is to use computational analysis of population proxies to directly model demographic trajectories before and after the K-Ah disaster. Our statistical models suggest unexpected demographic outcomes, enabling us to critically evaluate different human response scenarios that have been widely debated in the research literature. Equipped with an empirically grounded understanding of the multiscale impacts and legacies of the K-Ah eruption, we evaluate currently available archaeological evidence in light of our analytical outcomes. This enables us to tentatively infer a range of social-ecological processes that likely characterized human responses to the disaster, including identification of plausible pathways for future research. We conclude that quantitative reconstruction of demographic trajectories offers a powerful heuristic framework for tracking the complexity and contingency of human responses to major natural disasters, creating hypotheses and empirical foundations for larger research programs that aim to reconstruct a more comprehensive set of historical mechanisms.

**Fig. 1.**
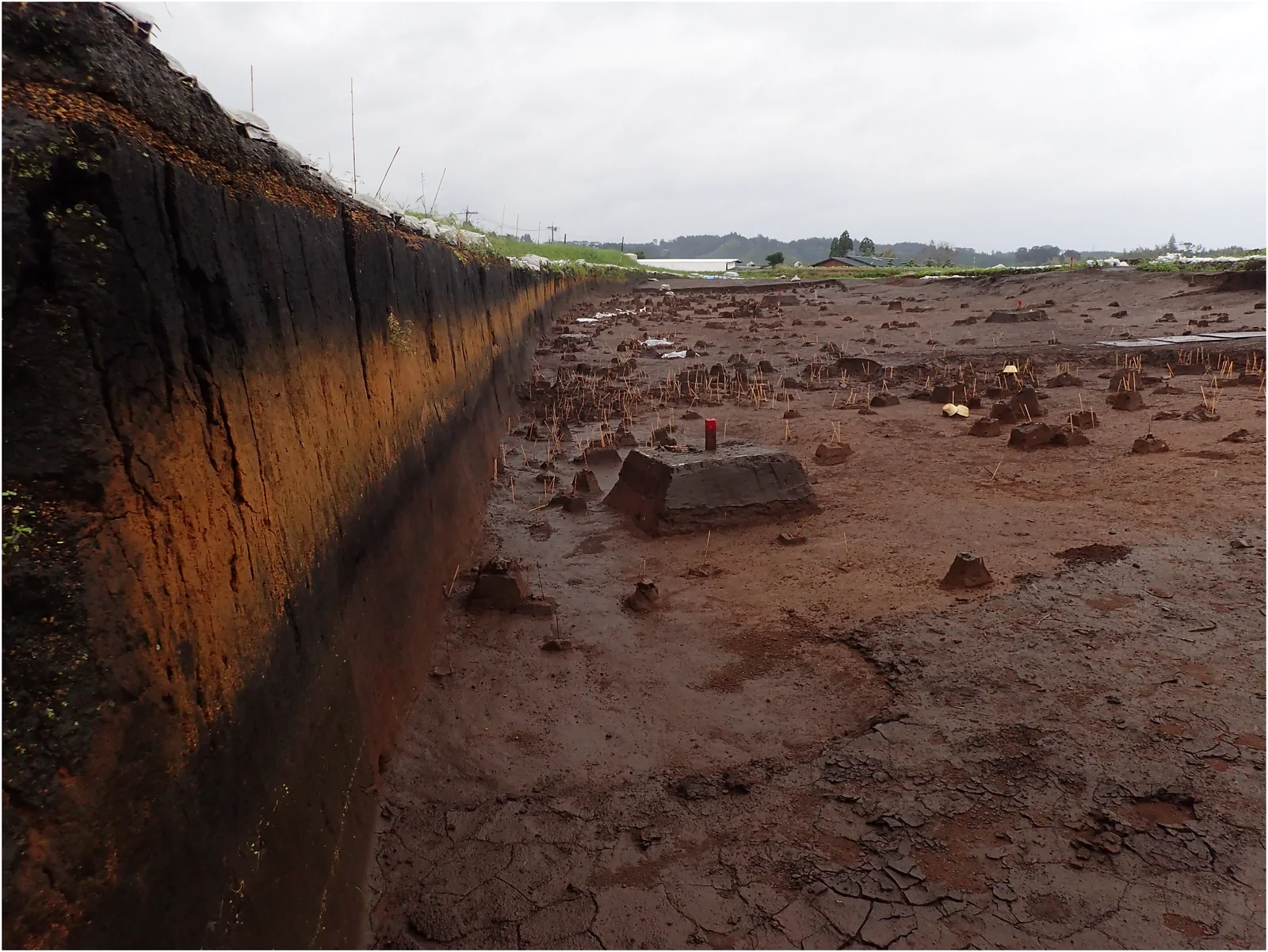
K-Ah volcanic deposit in southern Kyushu. This distinctive tephra layer offers a precise chronological “disaster horizon” across Western Japan. This stratigraphic cross section is from the Aihara archaeological site, located only 150 km from the eruptive epicenter. The local landscape experienced very heavy ashfall, likely ranging between 1000 and 2000 mm in total depth (see Fig. 3). The heavily compacted layer still averages 400 to 500 mm in thickness, with a shallow pumice layer overlain by thicker tephra deposits. Photo taken by the authors.

Given the accelerating impact of climate change on the contemporary world, there is renewed scientific and societal interest in understanding the ways in which human populations respond to sudden shocks, impacts, and disasters. Adopting a longer-term perspective, archaeologists have intensively debated the likely social, cultural and demographic consequences of sudden environmental change. Given their abrupt onset, massive impacts, and wide-ranging consequences for people, landscapes, and ecosystems, major volcanic eruptions continue to play a central role in archaeological debates about the factors that cause certain cultures to collapse and vanish, whereas others survive the impacts, persist, reorganize, and eventually rebound (*2*–*4*).

Archaeological research into the human consequences of major volcanic events is strengthened empirically by the fact that eruptions eject isochronic tephra deposits over large areas, establishing a precise chronological “disaster horizon” that can be studied at different spatial and temporal scales. Across diverse case studies, core research questions generally pivot around the extent to which major volcanic eruptions operate as “forcing” mechanisms, either terminating entire cultures, or deflecting entire social-ecological systems over to other developmental trajectories. Conversely, researchers have also focused on the extent to which social-ecological systems exhibit the capacities to absorb sudden volcanic shocks, persist, rebound, and eventually recover.

Despite the potential for longitudinal archaeological datasets to situate the conditions and consequences of catastrophic events within long-term social-ecological trajectories, much previous research has tended to examine the immediate impacts or short-term outcomes of major eruptions. Inevitably, this approach encourages shocks and disasters to be assessed against relatively static cultural and ecological baselines. Moreover, it encourages the generation of post hoc narratives that commonly describe observed response patterns. In turn, these tend to come down to reliance on simplistic binary oppositions that highlight either abrupt societal collapse and the sudden disappearance of entire cultures, versus indications of persistence, rebound, and recovery. Last, and given the low chronological resolution of many regional sequences, volcanic impacts and responses are often framed as abrupt breaks in artifact styles or highlight sudden shifts in site numbers or settlement patterns to infer demographic collapse or continuity.

To address these gaps and opportunities in knowledge and understanding, we focus on tracking human demography as a primary parameter in the wider reconstruction of general social-ecological responses to major volcanic disasters. This is combined with a hypothesis-driven approach, enabling us to define expectations and scenarios from the outset and to rigorously assess these with empirical results. The Japanese archipelago offers an excellent arena for advancing this kind of research, providing access to some of the highest-resolution archaeological records available anywhere in the world. Located on the Pacific Ring of Fire, the massive K-Ah eruption formed one major event in a relentless series of volcanic shocks and disasters that confronted ancient populations. Given the constant exposure to natural geohazards, the Japanese archipelago offers unique opportunities to develop longue durée perspectives into contingent human responses to major environmental shocks and disasters.

A distinctive feature of the Japanese archaeological record is the persistence of dynamic broad-spectrum foraging societies deep into the Holocene. These thriving hunter-fisher-gatherer communities define the Jomon archaeological tradition, characterized behaviorally by fluctuating phases of mobility and sedentism, alternating reliance on storage-based economies, and several intervals of increased greater social complexity (*5*). Operating for millennia without reliance on specialized agriculture, flexible Jomon foraging strategies combined a focus on nuts and plants, medium game like boar and deer, and the exploitation of aquatic resources. Another definitive feature of the Jomon foraging culture is long-running pottery traditions. Decades of intensive research by Japanese archaeologists have established detailed pottery typologies that offer high-resolution chronologies to support the study of cultural diversity and transformation, including human responses to environmental shocks and disasters (Fig. 2).

**Fig. 2.**
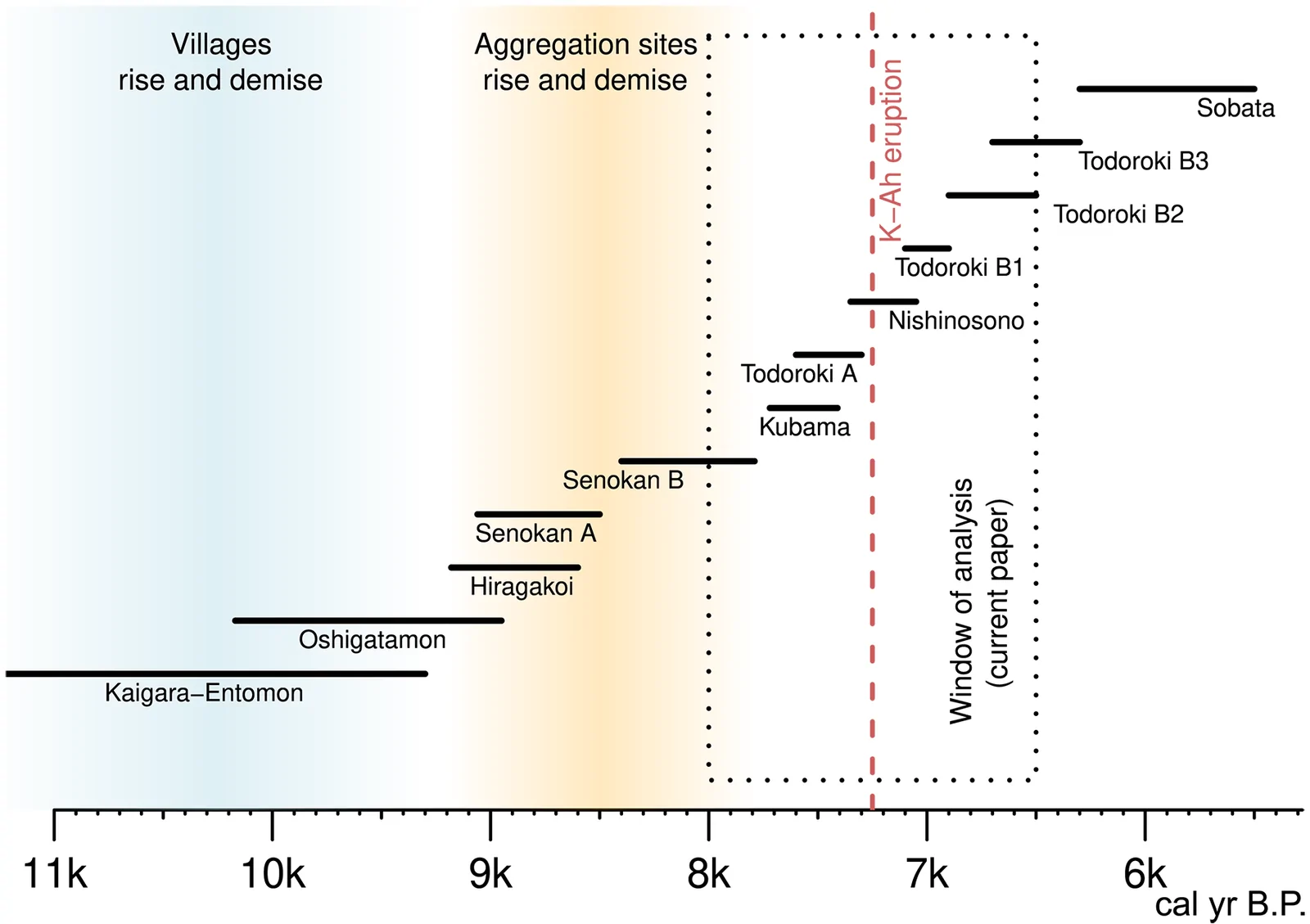
Main typochronological sequences in Kyushu.

Current archaeological consensus confirms that the Jomon tradition across Japan consisted of diverse local cultural groupings each with dynamic trajectories. There is abundant evidence that these local and regional communities underwent continuous cultural and demographic transformations, although the precise causes, conditions, and consequences remain disputed (*5*). The impact of major volcanic events features highly in many of these debates, none more so than the consequences of the massive K-Ah eruption, widely believed to have played a pivotal role in shaping the overall Jomon trajectory across Japan.

### The K-Ah volcanic eruption (7.3 ka)

Japanese geologists started reporting stratigraphic evidence for the K-Ah eruption in the 1970s (Fig. 1). With a volcanic explosivity index (VEI) of 7 (*6*), and a bulk tephra volume of 332 to 457 km^3^, the massive K-Ah caldera-forming eruption has now been reliably dated to 7300 years ago (*7*, *8*) and is widely acknowledged to rank among the largest ever Holocene eruptions anywhere on Earth.

The epicenter of the K-Ah eruption is centered on the Kikai caldera, a Quaternary volcano situated under the East China Sea, ∼30 km south of mainland Kyushu. Although the earliest geological history of the caldera remains poorly understood, Japanese volcanologists have identified at least two massive caldera-forming eruptions in this location, the first dated to 95 thousand years ago (ka), with the K-Ah forming the second major event at 7.3 ka (*9*).

The massive 7.3-ka K-Ah eruption should be understood as a punctuated sequence of related volcanic events rather than a single, sudden-onset catastrophe that affected Kyushu without prior warning (*9*). The overall chain of events followed a typical caldera-forming eruption sequence, with distinct plinian and climactic phases. K-Ah’s plinian phase has now been subdivided into three different eruptive events, each separated by at least two brief pauses, with the scale and intensity of eruptive impacts increasing over time. The first event was a small-scale to moderate-scale explosive episode, generating limited fall deposits. This was followed by a larger eruptive event that produced a thicker fall deposit, as well as localized pyroclastic density currents (PDCs). These PDCs, also known as pyroclastic flows, are fast-moving, surface-hugging surges of hot gas, ash, and rock. Typically traveling at hundreds of kilometers per hour and reaching temperatures of up to 1000°C, they form one of the deadliest volcanic hazards, affecting, eroding, and incinerating everything in their path. K-Ah’s third plinian event was much more powerful, generating widespread fall deposits, with increasing magma withdrawal leading to a large decompression in the magma chamber. After a further eruptive pause, this process culminated in the collapse of the magma chamber roof, initiating the onset of the climatic caldera-forming phase, which produced massive PDCs that surged at high speed over the sea and into the complex coastal and inland topography of southern mainland Kyushu (*9*). Additional impacts included massive tsunamis, powerful earthquakes, and thick deposits of volcanic ash (*10*).

### Differential volcanic impacts on terrestrial and coastal ecosystems

The precise impacts and legacies of the K-Ah eruption on surrounding environments and ecosystems require further research. Without doubt, Kyushu’s terrestrial ecosystems would have been severely damaged, with substantive evidence that the lush subtropical forest cover was abruptly replaced by extensive fields of unstable volcanic ash, especially in the southern areas of Kyushu that were most directly exposed to PDCs and the heaviest ashfall. The general mode and tempo of terrestrial ecosystem recovery correlate broadly with the overall depth of ashfall (see below). If the ashfall is below 25 mm, the soils survive, and vegetation can often recover within a season. However, heavier ashfall can have decadal-scale to centennial-scale impact because entirely new soils need to form. Over time, barren volcanic “wastelands” are slowly recolonized by pioneering plant species, followed by establishment of patchy open grasslands, and later the return of closed-canopy forests (*9*, *10*).

Across Kyushu, topographic and climatic factors would add further local variability, with steep slopes potentially shedding ash deposits quite quickly after heavy rainfall, resulting in secondary ash deposits in valley bottoms, floodplains, and river mouths. Vegetation on steeper slopes could then recover quite quickly, possibly supporting local fauna, whereas enduring ecosystem damage would become concentrated along valleys and open floodplains. In turn, major drainage systems would become increasingly choked with ash, resulting in water acidification, damage to fish spawning beds plus major disruption of food webs. The combined impacts on inland fisheries would have been severe, likely requiring at least decadal-scale recovery times (*11*). In contrast, Kyushu’s coastal and marine ecosystems would have been affected by tsunami damage and pumice rafting but could probably have bounced back within months to a few years given the dynamic flushing of ocean currents and massive nutrient fertilization resulting from volcanic ash (*12*).

Across Kyushu, these combined impacts would probably have initially had catastrophic general consequences for the overall availability of human food resources (*13*). However, substantial variability in local impacts, including exposure to PDCs, severity of ashfall, and subsequent redeposition, topographic, and elevation factors, all combined with strongly differential recovery rates for different kinds of ecosystems, could have resulted in a major spatiotemporal reorganization of key human food resources. Rapid recovery of coastal ecosystems, along with more enduring damage inland, would have likely created both challenges and new opportunities for surviving populations.

Although post-disaster ecological legacies within Kyushu were likely complex, the wider environmental impacts of the K-Ah eruption across the rest of the Japanese archipelago are not well understood, despite the fact that the diagnostic tephra provides a reliable chronological marker in several key paleoecological archives, including the Lake Suigetsu cores (*8*). Beyond Japan, there is growing interest in understanding potential impacts of this massive eruption on hemispheric and global climates. Recent studies suggest that the eruption could have triggered a multidecadal cooling episode, which, in turn, could have had consequences for ecosystems across Japan, possibly decreasing human food resources, although these possible outcomes require more detailed research (*14*).

### Cultural impacts of the K-Ah disaster on Jomon foragers?

As the enormous magnitude of the K-Ah eruption started to emerge, Japanese archaeologists were quick to consider the cultural implications of the disaster on local Jomon foraging communities. Decades of ensuing debate have pivoted around archaeological scenarios of abrupt societal collapse versus local survival (*10*):

1) Scenario 1: “Jomon Apocalypse”: More traditional archaeological interpretations directly correlate K-Ah impacts with the abrupt demise of the large village-based forager communities that had emerged in southern Kyushu substantially earlier than other parts of Japan (*15*). According to this scenario, sedentary communities thriving under the Holocene Climatic Optimum were immediately annihilated, producing a centennial-scale occupation hiatus. Supporting archaeological evidence includes the sudden termination of major pottery traditions just under the K-Ah tephra, combined with the appearance of new ceramic styles directly above it, usually assumed to indicate groups from distant areas moving into the empty landscapes. Although the devastated environments were slowly resettled, the lingering ecological damage ensured that Kyushu could never fully regain its earlier cultural dynamism. Instead, the main locus of Jomon cultural development was displaced from Kyushu over to eastern Japan. According to this apocalyptic scenario, the enormous environmental shock of K-Ah operated as a powerful “forcing” mechanism, directly determining the subsequent course of Japanese prehistory [e.g., (*16*, *17*)].

2) Scenario 2: “Jomon Survival”: Cumulative improvements in the resolution of archaeological and chronological data in Kyushu are now supporting revisionist perspectives on the cultural impacts of K-Ah. Reassessment of the major pottery groupings in relation to the K-Ah tephra now confirms clear stylistic continuity across the completed disaster cycle (Fig. 2). Specifically, the distinctive *Nishinosono* pottery tradition continues right through the K-Ah tephra, even at sites in southern Kyushu, likely indicating the partial survival of some local communities, even in areas located quite close to the epicenter (*18*, *19*). These emerging insights pivot archaeological interpretations over to the alternative themes of survival, continuity, and eventual recovery, highlighting the capacity for local societies to endure massive environmental shocks and eventually rebound. Although the precise demographic role of these small surviving groups in the eventual repopulation of Kyushu after K-Ah remains unclear, the archaeological confirmation of local cultural continuity minimally indicates that the human legacies of the eruption were more complex, variable, and contingent than assumed under the older apocalyptic scenario (*20*).

More recently, our research group has been developing a multiscalar understanding of the K-Ah catastrophe in southern Kyushu, situating the impacts of the massive eruption within a longer-term culture-historical trajectory (*21*). We use the heuristic concept of “social-ecological systems,” which avoids replicating binary “collapse” versus “survival” scenarios or resorting to simple explanatory arguments assuming environmental determinism (Scenario 1) versus cultural resistance (Scenario 2). Deploying this perspective, the K-Ah eruption can be embedded within deeper human-environmental interactions, with preceding conditions shaping later historical outcomes. Thus far, we have used this approach to reconstruct the dynamic social-ecological conditions that preceded the K-Ah catastrophe (*21*) and not affect a static cultural baseline. Instead, Jomon communities in southern Kyushu were already undergoing a series of dynamic transformations. These fluctuations had started long before the eruption and involved repeated shifts between mobile and more sedentary strategies and between dispersed and more clustered settlement patterns (Fig. 2). More generally, these preceding cumulative transformations were driven by other social-ecological processes and appear to have been shaped by contingent human responses to different kinds of environmental change, each with different modes and tempos, including slower shifts in postglacial vegetation patterns, as well as the impacts of shorter and more abrupt climatic downturns, including the 8.2-ka cooling event, which negatively affected deciduous forests and hence human plant food resources across coastal East Asia (*22*).

In terms of understanding human behavioral prelude to the massive K-Ah eruption, our research also indicates that the large Jomon villages of southern Kyushu had already been abandoned several millennia earlier (contra Scenario 1). Jomon communities had then shifted over to more mobile strategies, before returning to more clustered settlement patterns, marked by the rise of large ring-shaped aggregation and feasting locales, although use of these sites also peaked and eventually disappeared. Last, southern Kyushu was again inhabited by highly mobile foraging groups, occupying ephemeral sites within dispersed settlement strategies, resulting in a contingent cultural baseline that was suddenly affected by the massive K-Ah eruption (*21*).

### Four social-ecological expectations

Clearly, a more comprehensive social-ecological understanding of the entire K-Ah disaster cycle still lies some way ahead. In the meantime, we focus our research efforts by making four general social-ecological expectations, taking into account the fact that environmental damage would have been most severe in southern Kyushu, pre-disaster southern groups were already highly mobile and probably “vulnerable” demographically, and that interior and coastal ecosystems likely recovered at quite different rates:

1) The mobile forager populations in southern Kyushu would either perish in situ or be displaced northward as “disaster refugees.”

2) Post-disaster populations would concentrate along northern Kyushu coasts.

3) “Source-sink” population dynamics would then emerge as pioneer groups slowly moved from northern coasts back into damaged southern environments.

4) Sustained demographic “packing” along northern Kyushu coasts may eventually have triggered the rise of new cultural phenomena within this area.

### Research design: Modeling demographic trajectories

Given the current levels of knowledge and understanding, our aim in this contribution is to identify ways to evaluate the currently competing narratives of collapse versus survival and to start to generate empirical insights that help us evaluate our general archaeological model. In turn, these efforts enable us to gain analytical traction on the deeper issue of how ancient societies responded to massive environmental shocks. We tackle these challenges with an explicit, hypothesis-driven approach, defining specific expectations a priori and enabling us to track the operation of key parameters over the completed K-Ah disaster cycle. We deliberately target human demography as our primary analytical focus. This offers an independent line of evidence, enabling us to build on more traditional archaeological approaches to the K-Ah disaster, which have generally focused on understanding patterns of continuity and change in pottery sequences and other diagnostic material culture traditions. We also seek to place debates about the consequences of K-Ah within Kyushu into a much wider geographic context, focusing on regional-scale population trajectories across the entire Japanese archipelago.

### Explicit hypotheses

Working with the archaeological debates examined above, we formulated four testable predictions pertaining to demographic conditions and eventual consequences of the K-Ah disaster:

H1: Populations were growing across Japan under the Holocene Optimum.

H2: The K-Ah disaster severely reduced population levels within Kyushu.

H3: Surrounding regions of Japan were not affected by K-Ah, and populations continued to grow.

H4: Regional demographic outcomes correlate with the severity of K-Ah’s environmental impact.

To critically evaluate these four hypotheses, we inferred demographic trends across Japan before and after the K-Ah eruption using time-frequencies of radiocarbon dates and determined the extent to which any observed variation could be explained by the depth of volcanic ash deposit.

### Levels of inference

Given inherent limitations on space, combined with the preliminary nature of this research, we acknowledge that our exploration of human demographic perspectives on the K-Ah eruption cannot be expected to deliver a comprehensive historical understanding of the entire disaster. Instead, our targeted contribution operates over three distinct levels of inference:

1) Level 1: Modeling of proxy population data to directly reconstruct multiscalar demographic trajectories; these demographic models resolve our four research hypotheses, producing previously unidentified linsights.

2) Level 2: Spatially explicit demographic reconstructions are combined with independent archaeological information to hypothesize likely cultural processes and assess the veracity of our preliminary social-ecological expectations; this exercise identifies remaining gaps in data, knowledge, and understanding.

3) Level 3: Critical assessment of enduring knowledge gaps confirms plausible pathways for additional archaeological research; these future efforts can deliver a more comprehensive social-ecological understanding of the K-Ah disaster.

### Modeling regional-scale demographic outcomes

To model regional-scale variability in demographic responses, we examined spatiotemporal variations in the density of ^14^C dates. Although our central premises broadly align with an established tradition of studies interpreting the time-frequency of ^14^C dates as a proxy of changes in population size, settlement density, or, more generally, anthropic activities [see (*24*) for an extended methodological review], we argue that spatial variations in sampling intensity represent a key confounder that needs to be formally taken into account. Existing approaches either apply principles of relative risk surfaces to spatial density estimates by modeling overall sampling efforts (*25*, *26*) or use some form of permutation tests to identify local deviations from general trends in growth rates (*27*). Although both approaches address the impact of sampling efforts, they provide only a visual output (in the case of relative risk surfaces) or *P* values (permutation test) and, as such, do not yield estimates of effect sizes (i.e., local estimates of growth rates) nor do they offer a modeling framework that can incorporate predictor variables, a crucial component when considering the potential effect of rapid-onset extreme events on human activity.

In this contribution, we address these methodological challenges by extending the inferential approach introduced in (*28*, *29*). We first subdivided the four major Japanese islands into 61 hexagons (see Fig. 3), establishing a basic geographic framework to support regional-scale analysis of shared versus divergent demographic outcomes. We then fitted two Bayesian intrinsic conditional autoregressive (ICAR) models on the time-frequency of ^14^C dates 750 years before [i.e., 8000 to 7250 calibrated years before the present (cal yr B.P.)] and after (i.e., 8000 to 7250 cal yr B.P.) the estimated eruption date of K-Ah, set for analytical purposes to 7250 cal yr B.P. after (*8*). Sensitivity analyses to these time-window settings were carried out by comparing outputs based on narrower (500 years) and wider (1000 years) time-blocks.

**Fig. 3.**
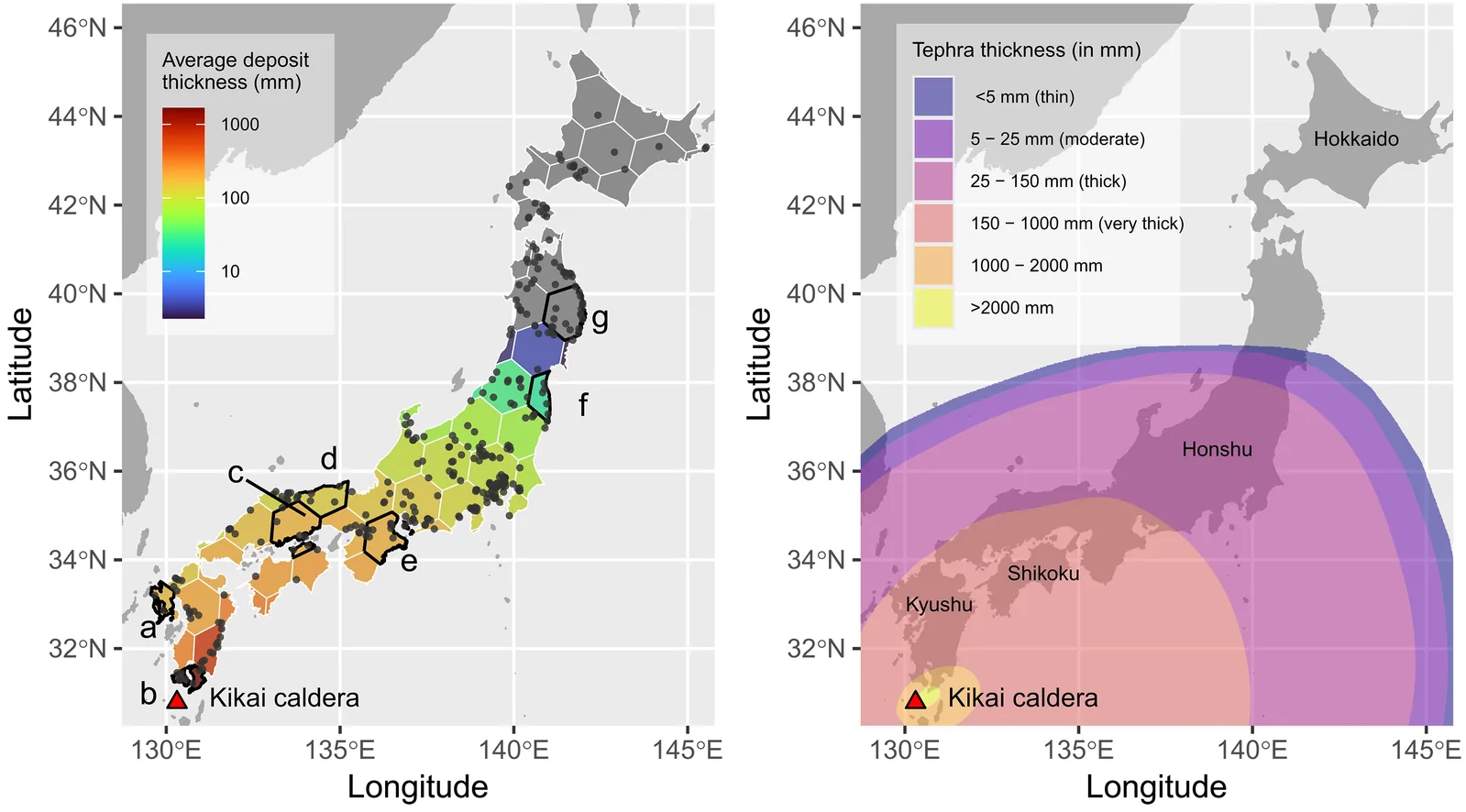
Map of the study area. (**Left**) Distribution map of the sampling location of ^14^C dates overlaid on the hexagonal areal units used to fit the Bayesian models; labeled areal units are discussed in the text. (**Right**) Tephra model deposit for the K-Ah eruption, after (*23*).

We considered two models:

1) Model A estimated the exponential growth rates before (parameter *r*_1,*i*_) and after (parameter *r*_2,*i*_) the K-Ah eruption for each hexagonal unit *i*.

2) Model B inferred the log odds ratio of the overall proportion of dates after the eruption (parameter η*_i_*), with negative values indicating a decline in the frequency of dates following the K-Ah event.

Last, both sets of parameters were spatially conditioned through the ICAR component of our model (i.e., adjacent hexagonal units were expected to have similar parameter values).

### Modeling severity and duration of ecological damage

To examine the extent to which regional demographic outcomes correlated with likely environmental impacts, we used ash depths as a first-order proxy. There is a general consensus that volcanic eruptions fall into a series of distinct types, although, in reality, the complex interplay of variables means that each event is unique in its outcomes and impacts. Despite this, hazards to human existence exhibit clear spatial patterning, ranging from more localized lava flows, earthquakes, and pyroclastic flows to global effects on climate (*13*, *30*). Important intermediate-scale threats include spatial patterning in proximal and distal ashfall, with the direction of the ashfall lobe determined by prevailing weather and wind conditions.

Accumulated ash affects local ecosystems differently depending on the depth. Lighter ashfall results in moderate burial of soils under 5 to 25 mm of tephra. Under these conditions, the soil is not deprived of oxygen or moisture and typically undergoes rapid recovery, usually completed by the next growing season. However, thicker ash layers produce longer-lasting damage because the buried soils are eventually deprived of oxygen, which kills off the soil biota and leaves a sterile ash layer. Ecosystem recovery can then be delayed by decades (25∼150 mm) to several centuries (>150 mm) because entirely new soils need to become established on unstable ash layers (*13*, *30*, *31*), although the exact nature of the response to the tephra load could vary considerably (*32*, *33*).

Given these parameters, the mapping of K-Ah ashfall patterns across Japan suggests clear spatial patterning in likely ecological impacts (Fig. 3). Using isopach models of the K-Ah tephra deposit (*23*, *34*, *35*), we identified that (i) the most severe ashfall (1000 to 2000 mm) was concentrated in the southern tip of Kyushu, including areas also affected by the pyroclastic flows; (ii) very thick (150 to 1000 mm) ashfall covered the rest of Kyushu and extended along a northeast axis into Shikoku and southern parts Honshu, although ashfall was lighter along the northern coast of Honshu; and (iii) thick ash deposits (25 to 150 mm) extended across western Japan, with minimal ashfall (<25 mm) in northern Tohoku and Hokkaido.

Using our general estimations of the severity and duration of likely impact on terrestrial environments, this spatial patterning in ashfall suggests centennial-scale environmental damage across Kyushu and large parts of the southern side of western Japan, with decadal-scale damage in surrounding areas. The northern (Sea of Japan) coast of western Honshu includes a long corridor of relatively lower levels of ash-mediated environmental damage, which extends to the islands located along the coast of northern Kyushu. This mapping of tephra fall across Japan examines likely damage to terrestrial ecosystems and does not assess impacts on coastal and marine ecosystems, which may have been able to rebound more quickly than inland areas (see above). Last, devastated outer islands in southern Kyushu, including Tanegashima and Yakushima, which were located close to the epicenter, would face additional challenges to environmental recovery as animal species that had arrived under lower Pleistocene sea levels (e.g., boar and deer) would need to cross open ocean to establish viable new populations.

To model spatial variability in environmental damage, we used the average thickness of the ash deposit within each hexagonal unit *i* as a predictor variable for *r*_2,*i*_ and η*_i_*. More specifically, we used a change-point model to account for the fact that existing studies summarized above suggest a nonlinear effect of ash deposits. All our parameter estimates (i) are conditioned by sampling intensity only in terms of precision (i.e., areas with a smaller number of dates would lead to less precise estimates), (ii) account for the spatial structure, (iii) offer insights on the magnitude of the differences in demographic trajectories, and (iv) the extent to which the amount of tephra deposit drove such variation.

## RESULTS

### Tracking demographic trajectories

Both Bayesian models identify intriguing demographic patterning in the prelude to and aftermath of the catastrophe. Model A uses the time-frequency of ^14^C dates to estimate population growth rates across Japan before and after the K-Ah disaster. Notably differences between the period before and after 7250 ka are identified. First, in the 750 years preceding the eruption, most regions of the archipelago exhibit positive growth rates, as predicted by H1. In contrast, Kyushu (and Hokkaido) exhibit weaker and possibly even negative values of *r*_1_ (Fig. 4, top left), albeit with high levels of uncertainty in the estimates (see also figs. S2 and S5). In Kyushu, this may signal that a population decline was already underway before the impact of the K-Ah eruption. As we noted above, populations during this interval had already shifted to more mobile lifeways.

**Fig. 4.**
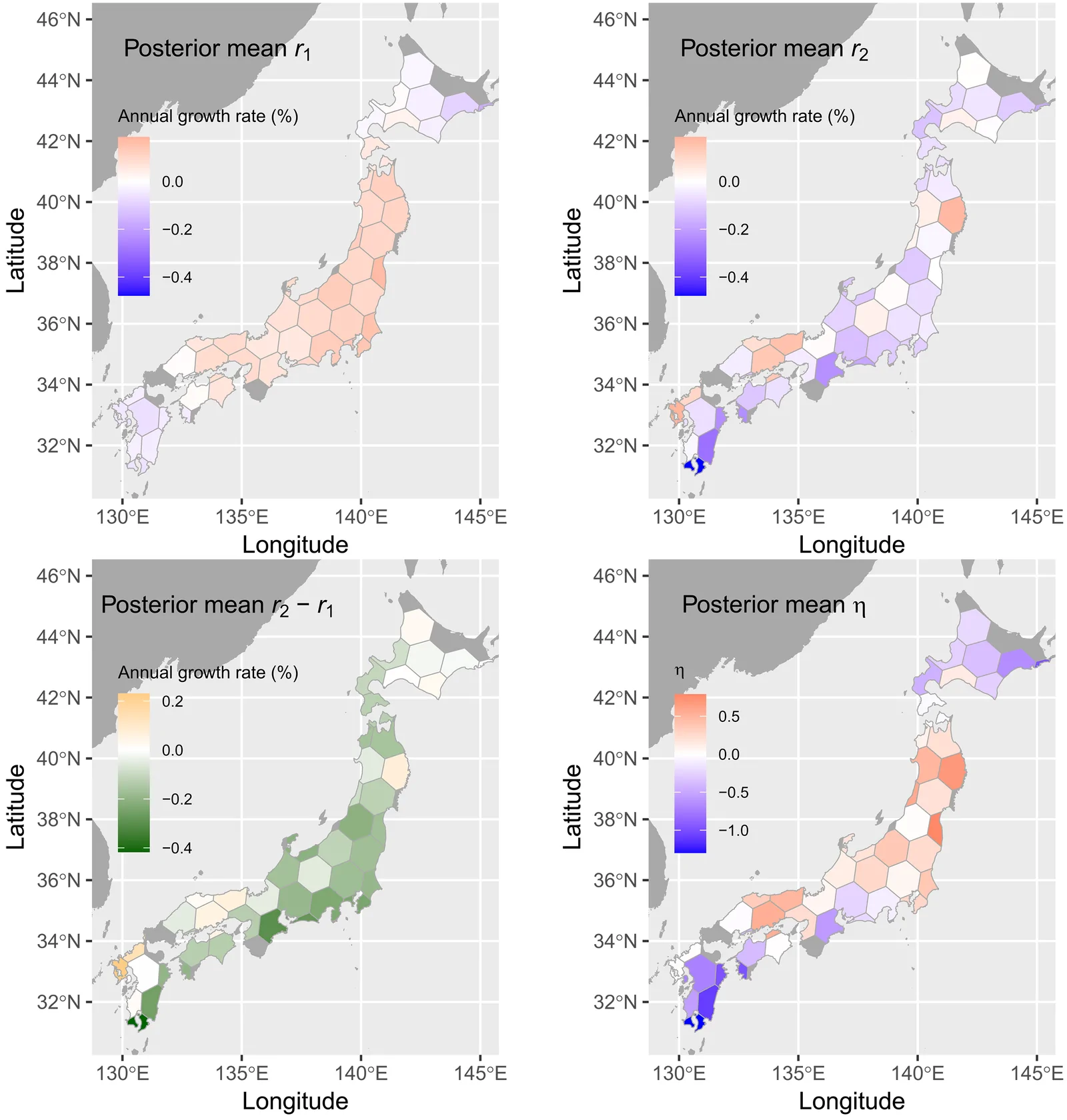
Posterior mean of *r*_1_, *r*_2_, and η. Mean posterior estimates for model A [*r*_1_ and *r*_2_ (**top row**); *r*_2_ − *r*_1_ (**bottom left**)] and model B [η (**bottom right**)] showing regional variations in demographic trajectories before and after the K-Ah eruption. See figs. S2 to S4 for the upper and lower boundaries of the highest posterior density interval. Regions with no samples, with estimates derived entirely from adjacent polygons, have been grayed out.

Second, regional growth rates after the eruption then show substantial diversity in the time-frequency of ^14^C dates. Most regions of the Japanese archipelago actually yield negative or close to zero *r*_2_ values (Fig. 4, top right; see also figs. S3 and S5), which marks a clear shift in demographic trends (Fig. 4, bottom left; see also fig. S5, bottom left), possibly indicating that the K-Ah event had negative population impacts well beyond immediate areas of the most severe damage, long assumed to be confined within Kyushu.

Model B offers a more direct insight into these regional-scale demographic fluctuations before and after the eruption. Across western Japan, this model picks up considerable regional-scale divergence in demographic outcomes, with both positive and negative estimates of η. Specifically, northern Kyushu and the northern coast of Honshu actually exhibit similar or even higher density of dates after the eruption, whereas other areas show a decline. In contrast, regions in eastern Japan have estimates of η that generally show that that the proportion of ^14^C dates from after the eruption were either the same or higher than those from before the eruption (Fig. 4, bottom right), with the notable exception of the island of Hokkaido (although the estimates in this region was associated with high levels of uncertainty; see also figs. S4 and S5).

These trends are still visible when at a narrower temporal window of analyses despite the reduced sample size, whereas the effects appear to be weakened at coarser chronological resolution when possible alternative demographic processes were probably in place (fig. S6).

To summarize, several predictions are met, but some other, more intriguing insights emerge:

1) Severe decline in local populations (to possible abandonment): Impacts were centered in proximal regions, primarily southern and central Kyushu, as evidenced by the largest decline in the proportion of radiocarbon dates {region b; *P*[*r*_2_(b) < 0] = 0.98 and *P*[η(b) < 0] = 0.97; Fig. 5}. However, these areas were possibly already undergoing population decline before K-Ah {*P*[*r*_1_(b) < 0] = 0.71}, and the eruption appears to have accelerated existing demographic trends {*P*[*r*_2_(b) − *r*_1_(b) < 0] = 0.97}.

**Fig. 5.**
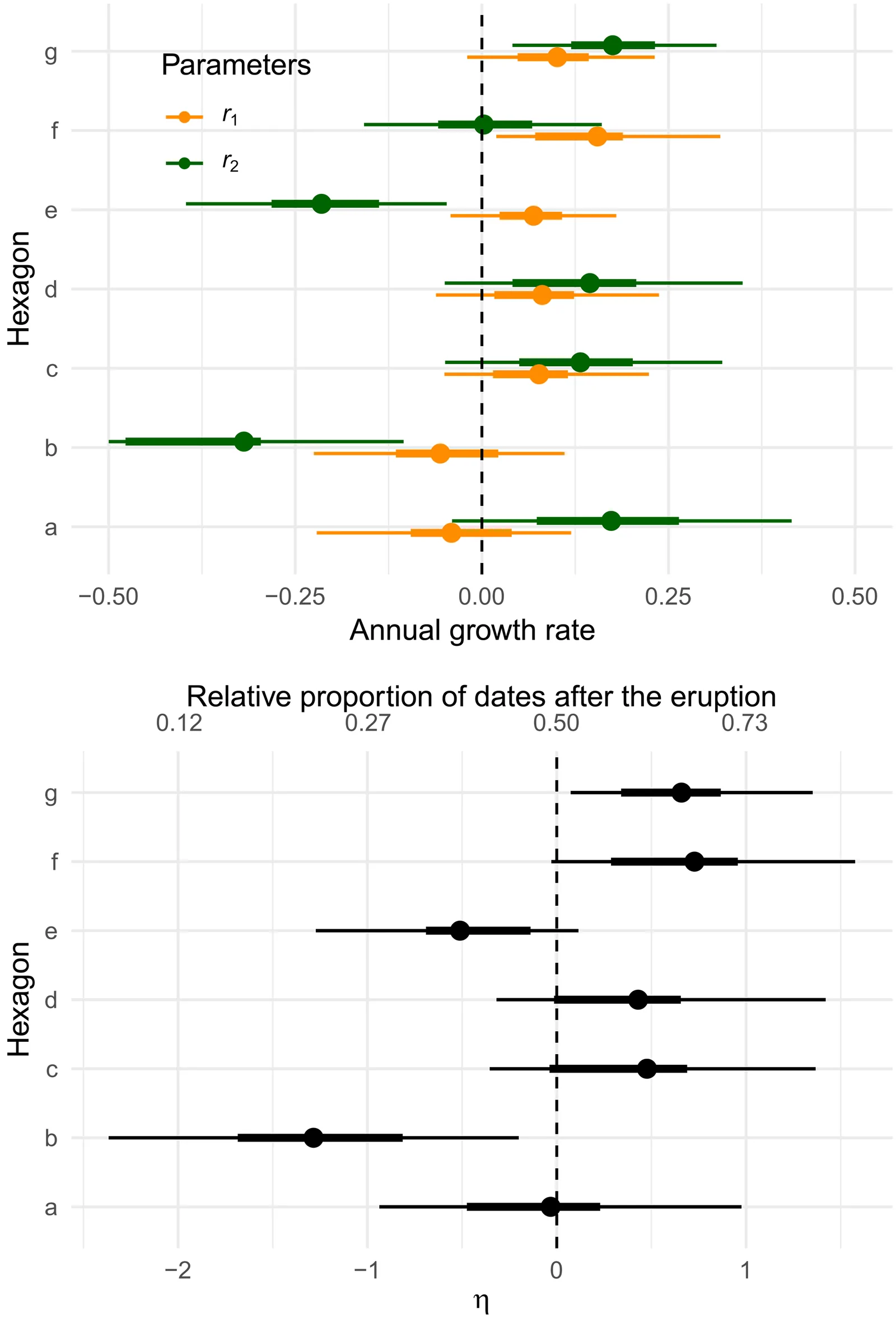
Posterior ranges of *r*_1_, *r*_2_, and η in key regions. Median, 50% (think line), and 90% (thin line) highest posterior density estimates of *r*_1_, *r*_2_, and η in selected hexagonal units (Fig. 3).

2) Distal impacts on regional populations across Japan. In contrast to overall demographic growth trends before K-Ah, some more distant regions of Japan (e.g., Ise Bay area; region e; Fig. 5) shift to downward population trends {*P*[*r*_2_(e) < 0] = 0.99, *P*[*r*_2_(e) − *r*_1_(e) < 0] = 0.98}, albeit with a smaller magnitude in the general decline of ^14^C density than areas noted above.

3) Local demographic resistance. Against predictions, populations in northern Kyushu exhibits invariance to disturbance in the form of continued growth, as evidenced by estimates of r_2_ and η (region a: *P*[*r*_2_(a) < 0.09] and *P*[η(a) < 0] = 0.52), and a similar trend emerges in northern Chugoku (regions c and d: *P*[*r*_2_(c) < 0.11] and *P*[η(c) < 0] = 0.17, *P*[*r*_2_(d) < 0.10] and *P*[η(d) < 0] = 0.17), suggesting either a maritime corridor of demographic resistance or population dispersal in some areas of western Japan. These regions managed to retain the positive growth rates exhibited before K-Ah (i.e., *r*_1_ ≈ *r*_2_), including both similar and a higher proportion of post-eruptive ^14^C dates (i.e., η ≥ 0). Our analyses also suggest that population trajectories in northern Kyushu (region a) are actually increasing after the catastrophe (i.e., *P*[*r*_2_(a) − *r*_1_(a) < 0] = 0.09) despite the close proximity to K-Ah (Fig. 5). However, the wide posterior range of η does not enable us to determine whether the overall density of dates changed. In contrast, several distal areas, including eastern Tohoku (regions g and f), exhibit similar demographic resistance (Fig. 5, *P*[*r*_2_(g) < 0] = 0.48, *P*[*r*_2_(f) < 0] = 0.01), although this is more predictable given the distance from the K-Ah epicenter.

### Environmental damage and demographic outcomes

Our model posteriors show no association between demographic trends and ashfall for lower average deposit thickness (fig. S7; 90% HPDI of β_1_: model A [−0.003, 0.001], model B [−0.934,0.419]) and weak negative effect for model A (fig. S7; 90% HPDI of β_1_+β_2_: [−0.01, −0.001]) at thicker deposits (i.e., above the change point; 90% HPDI of κ: [69 mm, 146 mm]). Results of model B do seem to suggest similar differences above the change point (90% HPDI: [64 mm, 137 mm]) were in place, although the posterior uncertainty was far wider (fig. S9; 90% HPDI of β_1_+β_2_: [−2.17, 0.3]) to suggest any clear support. Model comparisons with alternative model implementations with and without covariates also suggest no stronger support for this or any other variants (see tables S1 and S2), indicating that, for both models A and B, the impact played by the ashfall remains inconclusive. This could be in part not only due to limited statistical power (there is only a handful of regions with very large amount of tephra deposits, which happens to be also characterized by a smaller number of ^14^C dates and hence larger uncertainties in the estimate of demographic parameters) but also by the complexity of demographic trajectories that cannot be easily reduced to “thicker deposit = smaller growth rate” for all regions.

Nonetheless, some of the model outcomes were largely predicted:

1) Proximal regions suffering greater damage experienced higher demographic impact. As expected, the impact of tephra depth on *r*_2_ and η is more pronounced in proximal areas of southern and central Kyushu, where the ash thickness exceeds 150 mm, likely resulting in centennial-scale damage to terrestrial environments (Fig. 6)

**Fig. 6.**
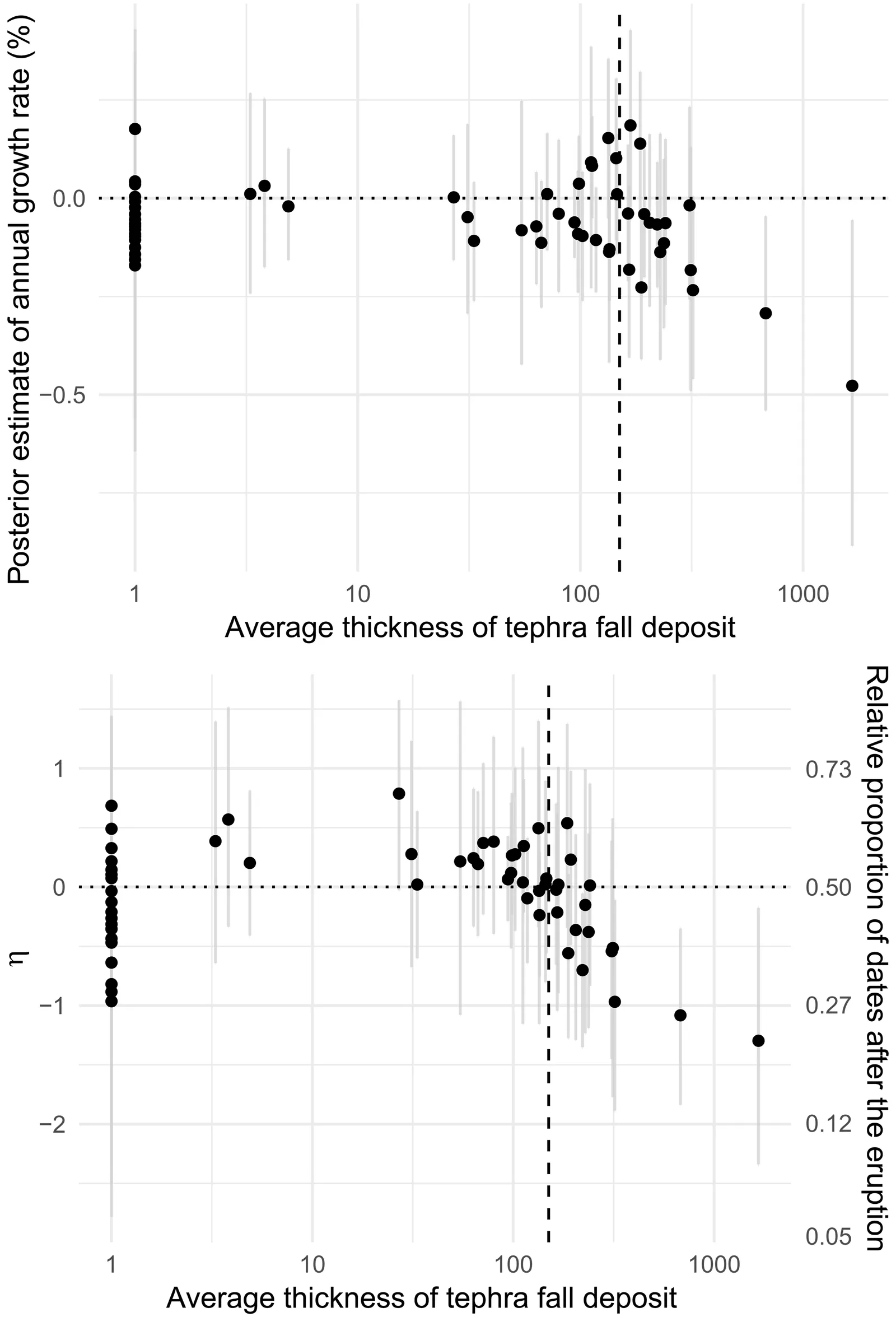
Relationship between average tephra deposit and demographic parameters. Scatterplot showing the posterior median and 90% highest posterior density interval of *r*_2_ (top) and η (bottom) against the average thickness of tephra deposit in each hexagonal unit. The dashed line indicates a tephra deposit thickness of 150 mm.

2) Distal regions with more limited damage underwent smaller and variable demographic change. Distance from the epicenter (>1000 km from K-Ah) may underwrite potential demographic resistance in far northern Japan (e.g., regions g and f) and/or relocation from regions more severely affected by the eruption.

Two additional insights indicate more contingent outcomes:

1) Spatial variability in environmental damage and demographic outcomes was possibly determined by the direction of the ash cloud. There are some weak statistical signal that suggest that damage to intermediate regions (i.e., <1000 km from K-Ah) appears to have been influenced by the axis of primary ashfall over Japan, which exhibits a clear east-north-east directional trend, and would have been produced by prevailing weather patterns at the time of the eruption. This factor may explain differences in the r_2_ and η estimates for regions closer to the volcano (Fig. 3). For example, there is greater damage in Shikoku than would be expected based on simple geographic distance from the caldera. The correlation between demographic trajectories and tephra thickness is in part demonstrated by the negative estimates of β_1_+β_2_ in model A (see also Fig. 6), although the signal for model B was weaker, and comparison with a model based on a pure geographic distance from the epicenter remains inconclusive.

2) Demographic resistance in proximal regions despite substantial terrestrial damage. Northwest coastal Kyushu and the northern coast of western Japan (i.e., the modern Shimane and Tottori prefectures) (see areas a, c, and d in Fig. 1) are located at the 150-mm transitional zone between “very thick” to “thick” ash depth (i.e., >1500 mm versus only 25 to 150 mm), suggesting impacts closer to multi decadal-scale damage, which would have been much less relative damage than adjacent regions located further south. These three areas experienced population growth after the K-Ah disaster (*P*[*r*_2_ < 0] < 0.11), despite their relative proximity to the epicenter, whereas areas with similar ash depth endured population declines. At least two of these regions have easy access to coastal and marine resources (regions a and d in Fig. 1). Possible processes generating these divergent demographic outcomes include internal growth within local populations, movement into the area from less damaged regions, migration by “refugees” out of the most devastated areas (e.g., southern Kyushu and Shikoku), or some combination of these three processes.

## DISCUSSION

### Level 1 inference: Demographic models and hypothesis evaluation

Working at our first level of inference, the spatially explicit reconstruction of demographic trajectories enables us to evaluate our four research hypotheses. H1: Forager populations were growing during the Holocene Optimum. Our reconstruction of demographic trajectories using time-frequency analyses of ^14^C dates shows that regional population levels across Japan were already fluctuating before the disaster. Not all regions of Japan were experiencing demographic growth before the disaster, and southern Kyushu was already undergoing a population downturn, possibly rendering the mobile local populations even more vulnerable to the subsequent disaster. H2: The K-Ah disaster reduced population levels within Kyushu. Our results indicate divergent outcomes, pointing to more complex and contingent impacts, with the predicted severe population declines confirmed in southern and central areas, whereas the maritime northwest exhibits population growth that starts before the disaster and persists and perhaps even accelerates afterward, potentially due to survivors from southern and central Kyushu relocating northward. H3: Surrounding regions of Japan were not affected and populations continued to grow. Further divergent trends emerge. Greater demographic impacts were identified in Shikoku and further along the Pacific coast of Honshu, both areas not traditionally associated with K-Ah impacts but where populations appear to have declined under heavier ashfall. Overall population growth rates in Japan were reduced after the disaster, although populations in more distal regions, including Tohoku, continue to grow. H4 Regional demographic outcomes correlate with the severity of K-Ah’s environmental impact. The continued population growth in northwestern coastal Kyushu and along adjacent coastal Honshu does not align with this prediction. There are population growth trends before K-Ah and indications of demographic stability in the aftermath. Other human responses may have generated these unexpected trends, including the general movement of population from damaged interiors to coastlines offering more reliable food resources, combined possibly with migration of initial survivors out of southern and central Kyushu into northern coastlines. In contrast, other regions fit this prediction, with severe depopulation occurring close to the epicenter, with declining impacts as distance increases and ashfall depth decreases.

#### Limitations

We note that the veracity of these primary inferences is inherently limited by the relatively low data resolution, combined with the inherent complexity of the phenomena we are aiming to model. Our model does capture the inherent uncertainty in our inferential exercise, as testified by the large posteriors observed in some regions with limited sample sizes. In some cases, we are not just limited in our ability to offer precise estimates of population growth rates but even to determine with certainty that there was a positive or negative growth (see fig. S5). This limitation in our data, in combination with potentially regionally divergent processes, did not allow us to conclusively identify the ash deposit as the sole explanatory variable of differences in demographic trajectories (tables S1 and S2). However, for other regions, our approach was able to capture clear signals. Yet, even for these regions, it is worth noting two factors that we should acknowledge in our interpretations. First, at a local level, site locational preferences might have shifted to areas that are less intensively sampled and excavated (e.g., from lowlands to hilly areas) but could have potentially been favored (e.g., hilly areas erosion might have exposed pre-eruption soils that could have been used by surviving groups). Second, the thickness of the tephra deposit is a potential confounder as it can cause a reduction in the number of observed archaeological sites both as the result of a demographic catastrophe in the past and by reducing archaeological visibility in the present. Although the prominence of rescue excavation in Japanese archaeology means that surface visibility is not a key determinant of where archaeological investigations takes place, temporary locational shifts to areas that are currently less populated (and hence less sampled) is something that we ought to consider as an additional factor behind at least some of the patterns we observe.

Despite these inferential limitations, our analyses establish a spatially explicit demographic model that empirically reconstructs how Jomon foragers living in different parts of the Japanese archipelago were affected by one of the Holocene’s largest-ever eruptions. One of the most unexpected insights is the possible role of human agency in structuring contingent demographic outcomes, especially in the proximal areas that suffered major environmental damage yet maintained population growth trends. Likely human factors included strategic migration of survivors out of the damaged interiors of southern and central Kyushu, followed by their incorporation into northern coastal communities.

#### Resolving archaeological scenarios: Social collapse versus cultural continuity?

These emerging demographic insights now enable us to revisit the binary “collapse” versus “continuity” scenarios that have defined traditional archaeological debate. First, the demographic model partially aligns with the argument that K-Ah triggered a “Jomon Apocalypse,” at least in southern and central Kyushu, the remoter southern islands, and possibly parts of Shikoku and surrounding areas. All these regions suffered major damage, extensive depopulation, and, in several areas, an enduring cultural hiatus, with these demographic signals disappearing only when we consider a wider chronological window (fig. S6). In contrast, additional insights undermine the conclusion that K-Ah acted as a deterministic forcing mechanism that alone shifted the course of Japanese prehistory: The large Jomon villages had already disappeared (Fig. 2), whereas our demographic modeling confirms that differential population trajectories characterized northern and southern Kyushu before the disaster and that they also persisted after it, indicating that additional demographic and cultural factors were also in operation. Last, our model confirms that K-Ah also had diverse demographic impacts in other parts of the Japanese archipelago.

Likewise, the “Jomon Survival” scenario demands further evaluation and refinement given the evident complexity of post-disaster demographic outcomes. First, our population modeling indicates that any local survivors maintaining the *Nishinosono* pottery tradition in small southern and even central Kyushu refugia would have been operating in both damaged and severely depopulated environments. Given the inherent demographic vulnerability of the kinds of small and highly mobile groups that existed before K-Ah, the massive ecological impacts of K-Ah would have abruptly eroded general food security plus profoundly disrupted vital social networks. As a result, initial survival could easily have been followed by subsequent regional abandonment, driven either by growing food shortages or equally likely by the need to maintain links and alliances with the kinds of larger and more stable demographic units that existed along northern coastlines. Another intriguing issue is how local groups survived in the ash-decked landscapes, perhaps congregating along steeper slopes that had shed thicker ash or by operating along faster-flowing river systems that recovered more quickly. One key issue is how survivors were harvesting and processing post-disaster resources, including reliance on “emergency foods,” the role of traditional versus novel technologies, and not least the precise social, cultural, and even psychological role of the pottery-based cooking and consumption practices that had sustained the distinctive *Nishinosono* ceramic tradition through what must have been an abrupt and fundamental reorganization of both resource landscapes, mobility strategies, and wider social interactions. Minimally, these isolated and demographically fragile local groups would eventually have required a larger social world in which to operate, culminating in new social networks that extended into northern coastal areas. Over time, these post-disaster shifts in demography and social connectivity could have generated source-sink dynamics, with robust northern populations sustaining a more mobile and ephemeral human presence in central and southern Kyushu. This may also suggest that the eventual repopulation of Kyushu’s southern mainland and remote outer islands would have involved not only a complex and punctuated interregional process, possibly involving intrinsic local population growth, but also other kinds of demographic movement back into the worst damaged areas (*36*, *37*).

To summarize, neither of the binary collapse versus survival scenarios can fully accommodate the complex and contingent ways in which human populations responded to the K-Ah disaster. Clearly, variability in the preceding environmental, cultural, and demographic conditions were important in structuring subsequent social-ecological outcomes. Beyond the geography of volcanic impacts, and the differential recovery rates of diverse ecosystems, our models suggest that human agency also appears to have played a substantive role in decisions to migrate out of and back into damaged areas, demanding the forging of new social relations, and the innovation of local post-disaster economies. Understanding these human strategies requires returning to the archaeological evidence.

### Level 2 inference: Demographic patterns and independent archaeological information

Here, we combine our spatially explicit demographic reconstructions with a thematic exploration of archaeological evidence in Kyushu. The goal is to critically evaluate our four general social-ecological expectations (i.e., that surviving southern groups were displaced northward; post-disaster populations gathered along northern coasts; source-sink population dynamics emerged in the resettlement of southern Kyushu; sustained population in northern coastal Kyushu triggered the rise of new cultural phenomena), enabling us to hypothesize potential cultural processes and identify remaining gaps in data, knowledge, and understanding.

#### Theme 1: Pre-disaster demographic “fragility” in southern Kyushu?

Available archaeological evidence appears to align with our demographic modeling, and confirms that southern Kyushu was quite sparsely populated before K-Ah, with small bands relying on high mobility and long-range social networks to buffer risk. New patterns of cultural diversity were emerging across Kyushu before the K-Ah eruption, with distinct southern and northern trajectories. In southern Kyushu, a more mobile adaptation emerges from the start of the *Todoroki* pottery group (Fig. 2). Occupations during the *Todoroki A* pottery phase are predominantly ephemeral camps, with arrowheads displacing heavy (plant) grinding equipment in tool kits, possibly indicating the growing importance of hunting. Long-range cultural influences are also in evidence in the new pottery styles, and appear to emanate from Central Honshu, located some 900 km away to the northeast (*18*, *21*). Additional traits reaching southern Kyushu from this area include slit stone earrings (*38*–*40*). These items of personal adornment appear to have continental origins and expanded into the Japanese archipelago from the north. Before K-Ah, their distribution within Kyushu is limited to southern areas, and no examples have been recovered from northern sites until after the disaster.

#### Theme 2: Robust pre-disaster populations in northern coastal Kyushu?

Northern coastal Kyushu exhibits a different archaeological trajectory immediately before K-Ah, with indications of increasing reliance on maritime resources. Evidence for marine mammal hunting appears at northern coastal sites including Tsugume-no-hana (Hirado City, Nagasaki Prefecture), with stone harpoons entering tool assemblages, part of a wider trend toward maritime intensification (*41*–*43*), possibly driving the population growth trends identified by our demographic modeling.

#### Theme 3: Post-disaster migration and social interaction networks?

Our demographic modeling confirmed population growth continued in northern coastal Kyushu after the eruption. This population trend may have been boosted by refugees abandoning southern Kyushu and moving northward. Likewise, the major population decline in southern Kyushu may have been driven by massive in situ fatalities combined with strategic migration to safer areas in the north. Archaeological evidence lends preliminary support to either northward migration, the forging of previously unobserved kinds of interaction networks across post-disaster Kyushu, or a combination. Before the eruption, small numbers of slit stone earrings have been recovered from a few southern sites. Directly after K-Ah (*Nishinosono* pottery phase), the overall number of earrings in circulation expands, plus they start to appear for the first time at northern coastal sites (*39*, *40*), with their exchange possibly important in negotiating post-disaster networks and alliances. However, this interpretation relies on typochronological analysis of earring distributions in relation to the K-Ah tephra, which is much thicker in southern Kyushu, making stratigraphic interpretations more robust than in northern Kyushu, where a thinner tephra, especially at sites with disturbed contexts, adds additional uncertainty. Over time, the distribution of the slit stone earrings expands across western coastal Japan, and eventually over into southern coastal Korea, hinting at the expansion of long-range interaction networks with regions whose environments were less badly damaged by the K-Ah eruption.

#### Theme 4: Protracted “source-sink” population recovery dynamics in southern Kyushu?

Our demographic modeling confirmed falling population levels in southern Kyushu before K-Ah and major declines afterward. The post-disaster demographic recovery of southern Kyushu after K-Ah falls into the *Todoroki B1* to *B3* pottery phases (Fig. 2). At the moment, it is unclear whether this wider repopulation process was driven primarily by southward migration from northern coastal areas or via local demographic rebound among southern survivors who had maintained the *Nishinosono* tradition or a combination of the two. Given the scale and duration of interior environmental damage, we predicted the emergence of source-sink population dynamics after K-Ah, with the robust demographic units in northern coastal Kyushu providing small mobile groups that were involved in the eventual resettlement of southern Kyushu. Archaeological evidence aligns with these expectations, suggesting that demographic recovery in southern Kyushu was a protracted and possibly punctuated process, with post-disaster groups reliant on northern connections. The Numerikawa No. 2 site, located in southern Kyushu, illustrates these patterns. Occupation starts in the *Todoroki A* phase and is then overlain by the thick K-Ah tephra (Fig. 2). The *Nishinosono* “survivor” pottery is absent, suggesting no in situ survival at this site. People return in the *Todoroki B1* phase, and the site is then occupied continuously to the *Todoroki B3* phase (*44*). Obsidian procurement strategies shift over time. During the *Todoroki A* phase, 84 of 107 obsidian artifacts can be sourced back to deposits in northern Kyushu over 200 km away, confirming that southern groups already maintained exchange networks with northern areas before K-Ah. In contrast, the groups that returned to the site after the catastrophe relied entirely on northern obsidian sources (27 obsidian artifacts)[(*44*): tables 9 and 13]. Evidence from site occupations also suggests that post-disaster populations developed new adaptive strategies to cope with the damaged environments, primarily a greater reliance on mobile hunting (*20*). Before K-Ah (*Todoroki A* phase), there was greater investment in site-based activities, including construction of numerous stacked stone hearths, with 20% of the lithic assemblage consisting of heavy grinding stones possibly used for processing nuts or plants. After people eventually return (*Todoroki B1* to *B3* phases), the activities are more ephemeral, with only one stacked stone pit. The tool assemblage is dominated by scrapers, possibly for processing animals, whereas the heavy processing tools are absent. Over time, these mobile populations would have expanded back into the remote offshore islands located close to the epicenter of the K-Ah eruption, a process requiring new adaptive strategies, possibly including the deliberate “re-stocking” of damaged island ecosystems with wild game (*20*).

#### Theme 5: Post-disaster maritime intensification in northern coastal Kyushu?

Our modeling indicates that populations in northern coastal Kyushu continued to grow through the K-Ah catastrophe, probably via internal demographic processes, possibly augmented by refugees vacating southern Kyushu. Internal demographic growth aligns with the archaeological evidence, which highlights that maritime intensification continued and perhaps even accelerated after the catastrophe (*Todoroki B1* to *B3*). After K-Ah, the number of stone harpoon tips increases further, possibly in the *Todoroki B1* phase and certainly from *Todoroki B2* and into the following *Sobata* period (Fig. 2). The geographic distribution of harpoon heads also expands out of coastal northern Kyushu after the disaster, reaching the Gotō Islands located 40 to 80 km offshore, as well as Okinoshima island 80 km to the north [e.g., (*42*)], an expansion possibly driven by groups seeking new locations for sea mammal hunting. Maritime exchange networks also become more important after K-Ah, with northern Kyushu offering diverse mineral resources, including talc used as a decorative pottery temper [e.g., (*45*)] plus the intensified exchange of obsidian derived from sources across northern Kyushu [e.g., (*46*, *47*)]. Overall, maritime intensification appears to correlate with sustained population growth, including possible demographic dispersals into new areas and the rise of larger maritime interaction spheres.

#### Theme 6: Post-disaster outcomes drive new cultural expansions?

Our fourth social-ecological prediction is that continued demographic “packing” in northern coastal Kyushu may have generated distinctive new cultural phenomena. One plausible candidate for this kind of outcome is the *Sobata* tradition, which emerges within the maritime “core” area of northwest Kyushu directly after the *Todoroki B3* phase (Fig. 2). Typologically, the *Sobata* pottery phase is defined by diagnostic talc-tempered pottery, which gives vessel surfaces a characteristic shimmering appearance. Archaeologists agree that the *Sobata* phenomenon is defined by intensified exploitation of coastal and offshore resources, coupled with participation in an expansive maritime interaction sphere. The *Sobata* tradition emerged in a very localized area of coastal northwest Kyushu and then underwent rapid geographic dispersal over the following centuries, extending first along the Honshu coast, and then moving over into coastal Korea, culminating in a long-range maritime expansion into the extended Ryukyu archipelago [(*43*); e.g., (*45*, *48*)]. One plausible explanation is that the *Sobata* expansion was a demographic phenomenon triggered by continued post-disaster population growth in the “core” area of northwest coastal Kyushu, forcing off-shot groups to seek out new marine hunting locales in surrounding areas.

### Archaeological evaluation: Four social-ecological expectations

At this juncture we can return to our four preliminary social-ecological expectations: (i) Northward migration. Provisional archaeological support for the relocation of demographically “vulnerable” southern groups into northern areas is provided by post-disaster expansion of the slit stone earrings into new areas; exchange of these symbolic artifacts may have supported negotiation of new kinds of interregional social relations. (ii) Populations gather along northern coasts. Evidence for continued demographic growth in northern Kyushu after K-Ah aligns with clear archaeological evidence for post-disaster maritime intensification along northern coasts and islands. This trajectory originates before K-Ah and may accelerate in the aftermath, although there may be some initial post-disaster delays before the tempo resumes. Northward expansion of the slit stone earrings may signal additional migration to northern coasts. (iii) “Source-sink” dynamics emerge across post-disaster Kyushu. Archaeological evidence points to the novel subsistence practices, high mobility, and ephemeral presence of post-disaster groups in southern Kyushu, combined with an apparent post-disaster reliance on northern networks (expansion of slit stone earrings) and raw material sources (obsidian). Given the severe and sustained population contraction identified in southern areas after K-Ah, it appears likely that these mobile post-disaster “pioneer” groups relied on the larger demographic units in the north to establish a viable presence further south, although the precise environments attracting and supporting these mobile groups remains unclear. Possibly, returning and rebounding game populations (boar and sika deer) were an obvious target. (iv) Impact of population packing along northern coasts. Archaeological evidence confirms that maritime intensification in northern Kyushu was coupled with the demographic growth trends that persisted throughout the K-Ah catastrophe cycle. The new *Sobata* cultural tradition that emerged directly out of these northern demographic units and then spread, possibly via demic diffusion, into surrounding maritime niches was probably the culmination of this deeper historical trajectory. However, in channeling post-disaster population movements from interiors to coastlines and from southern areas into northern Kyushu, the K-Ah catastrophe may only have “accelerated” an existing process rather than initiating it. To summarize, combined demographic and archaeological evidence lends preliminary support to our four social-ecological expectations while generating a suite of hypotheses about likely cultural mechanisms and processes and identifying enduring gaps in knowledge and understanding.

### Level 3 inference: Identifying plausible pathways for future research

We now move into our third level of inference, which aims to identify plausible pathways for future research into the K-Ah eruption, focusing on proximal (Kyushu), distal (Japanese archipelago), to potential global-scale impacts. These efforts generate a multiscalar research agenda that will deliver a more comprehensive understanding of the entire K-Ah phenomenon.

#### Investigating proximal impacts (Kyushu)

A suite of priority research themes emerges in Kyushu: (i) We need a much higher-resolution multiscalar understanding of the environmental history of Kyushu across the K-Ah disaster cycle, especially the variability, chronology and topographic influences on local impacts and recovery times including the return of riverine fisheries and transitions from ash fields to open grasslands and closed-canopy forests, including the extent to which local refugia close to the epicenter could have supported small human and animal populations directly after the eruption. We also need to understand how cornerstone species like wild boar and sika deer survived, adapted their feeding patterns, and expanded back into damaged areas plus the shifting post-disaster availabilities of diverse plant and nut resources; (ii) we need to know how local survivors endured and adapted to the massive devastation in southern Kyushu, including impacts on human health and mobility, their behavioral adjustments, the new and traditional technologies they deployed, as well as the harvesting, processing, and sharing of foods, not least the role of the iconic *Nishinosono* “survivor” cooking tradition; (iii) we need to know how populations were able to expand back into southern Kyushu, exploring secondary abandonments via the potentially punctuated character of local settlement chronologies, and whether this entire process can be captured with source-sink population models; (iv) the potential of additional site-based proxies including high-resolution pottery chronotypologies (Fig. 2) to model this demographic recovery in southern Kyushu, focusing on the eventual resettlement of the most severely damaged areas including the remoter offshore islands like Tanegashima and Yakushima (e.g., was there a “wave of advance” from surrounding regions, “leapfrog” movements into optimal resource areas, or localized population rebound originating in surviving groups); (v) is the extent of additional archaeological, dietary, and culinary evidence for initial and intensified maritime exploitation along northern coastal Kyushu before and after K-Ah; last (vi) the extent to which the emergence and long-range expansion of the maritime *Sobata* tradition can be framed as a demographic phenomenon that was potentially initiated or accelerated by population-scale impacts and legacies of the K-Ah disaster.

#### Understanding distal impacts (Japanese archipelago)

One of the most unexpected results of our modeling was the identification of demographic consequences of K-Ah extending well beyond Kyushu, including a more general population slowdown across eastern and central Japan. Before K-Ah, most areas (and particularly Honshu Island) had experienced positive growth rates, possibly due to the stabilization of coastal ecosystems after millennia of sea level rise, combined with peaking temperature and humidity of the Holocene Optimum. After the K-Ah catastrophe, growth rates decreased in many places and, in some cases, even showed negative trends. Moreover, these impacts appear to have had sustained consequences over several centuries as our growth estimates are based on a 750-year average. In other words, a short-term (i.e., <100 years) disruption followed by a quick recovery would still have yielded positive growth rates, but this pattern was not observed. These unexpected population downturns in distal areas receiving lighter ashfall require further research.

Three possible factors may have been involved: (i) possible fluoride poisoning of people and animals in distal regions (*49*); (ii) the consequences of the smaller volcanic ash particles, which are most hazardous to lungs, being blown further into these distal areas, affecting the health of human and animal populations (*50*); and (iii) the demographic impact of accelerated tooth attrition on deer and wild boar populations as they grazed in ash-filled landscapes, in turn, reducing life spans and decreasing human food resources (*51*). Confirming the role of these three intersecting factors would require targeted osteological analysis of bone deformations and tooth wear patterns along the K-Ah ashfall transect, with the goal of comparing health traits and tooth attrition rates before and after the eruption, combined with particle size analyses of K-Ah ashfall deposits.

#### Exploring hemispheric to global-scale climatic impacts of the K-Ah eruption

One final under-researched impact of K-Ah pertains to potential climatic downturns operating across larger East Asian to northern hemispheric and global scales. This was one of the most powerful Holocene volcanic events (*14*), and abrupt multidecadal or even centennial-scale climatic excursions were triggered. Regional-scale to local-scale impacts of this climate change on human food resources and wider social-ecological systems await further research, with the K-Ah tephra offering a precise chronological marker across diverse cultural, demographic, and paleoecological archives.

### The K-Ah catastrophe: Toward a new social-ecological synthesis

After initial reporting of the enormous K-Ah volcanic eruption in the early 1970s, archaeologists have continued to debate the significance of this massive environmental shock for ancient people, societies, and landscapes. This paper uses a social-ecological perspective to generate human demographic models of the disaster’s diverse multiscale impacts and legacies. By operating across three different levels of inference, the paper does not aim to reconstruct a comprehensive understanding of the disaster in all its minute historical detail. Instead, it identifies a suite of expected and unexpected demographic outcomes that require further research to fully understand and explain. Demographic insights are combined with exploration of available archaeological data to confirm the general veracity of our social-ecological model: Jomon societies in Kyushu neither “collapsed” entirely nor simply “survived.” In this way, our current results strengthen the revisionist interpretation that deeper impacts and legacies of the K-Ah eruption cannot be reduced to a singular apocalyptic “forcing” mechanism that terminated vital regional trajectories and permanently displaced subsequent cultural dynamism to other parts of Japan. Viewed over the longue durée, ancient mega-disasters like K-Ah not only were clearly both enormously destructive in human and environmental terms but also generated local social-ecological responses that need to be understood as being complex, multiscalar, and contingent. Clearly, the impacts and legacies of K-Ah were certainly embedded in deeper trajectories of transformation, with the interaction of human agency and diverse ecosystem recovery times shaping eventual cultural, demographic, and environmental outcomes. Many research themes now emerge, including initial survivals and secondary abandonments, the operation of source-sink population dynamics, fluctuating exchange and interaction networks, the importance of innovation, memory and cultural traditions in sustaining post-disaster communities, the role of maritime affordances and long-range diasporas, as well as intersections between proximal, distal, and global-scale ecological impacts. In this way, further integrated social-ecological research into the entire K-Ah phenomenon looks well placed to advance the wider field of paleo-disaster studies. In turn, these empirical insights and deeper interpretive understandings engage with wider academic and societal debates about our deeper existential sustainability, including the capacity of diverse cultures and societies—in the past, present, and future—to withstand sudden shocks, impacts, and environmental catastrophes (*13*, *52*).

## MATERIALS AND METHODS

### Radiocarbon data

We collated ^14^C dates from the curated version of the radiocarbon database of the National Museum of Japanese History (*53*) for our analyses. We considered, from an initial set of over 42,000 dates, samples dated on materials of terrestrial origin with a cumulative calibrated probability density between 8000 and 6500 cal yr B.P. equal to or larger than 0.5. To account for the imbalance in sampling intensity, we considered samples recovered in close proximity in both space (<100 m) and time (<100 years) as an analytical unit and selected the sample with the smallest measurement error. We considered only sample locations from the four main islands of the Japanese archipelago (see further details in Supplementary Text). The final sample consisted of 540 ^14^C dates, although we also examined two separate subsets with different chronological windows (1000 and 2000 years) and sample sizes (*n* = 371 and *n* = 703) to assess the sensitivity to these settings (see Supplementary Text and figs. S1 and S6).

### Bayesian demographic models

We first subdivided our window of analysis into 61 hexagonal spatial units with a diameter of 125 km. This spatial scale was selected by trading off spatial resolution and sample representativeness. We then fitted two Bayesian ICAR demographic models on our ^14^C data. Our first model (model A) estimated exponential growth rates before (*r*_1_, 8000 to 7250 cal yr B.P.) and after (*r*_2_, 7251 to 6500 cal yr B.P.) the K-Ah eruption across 61 hexagonal cells covering the spatial extent of our window of analysis, following the same procedure adopted by Crema *et al.* (*28*). We modeled *r*_2_ using the log_10_ of the average depth of K-Ah tephra deposits in each cell, obtained from the ashfall/tephra fallout model published in (*23*). The ICAR component of our model accounted for spatial autocorrelation, enabling estimates of *r*_1_ and *r*_2_ of each cell to be partially informed by neighboring regions. Our second model (model B) used the same spatial structure and predictor but instead modeled the overall proportion of radiocarbon dates before and after, with the key parameter η equivalent to a log odds ratio. An estimated η of 0 would imply that the radiocarbon density before and after the eruption is comparable, whereas an η of 1 would mean that ∼26.8% of the dates in our time range are expected to be from before the eruption, suggesting an increase in the overall density. It is worth noting that the two approaches model changes in the radiocarbon density slightly differently. For example, if *r*_1_ *= r*_2_ > 0, then η > 1 as more dates will be expected in the latter interval under a constant positive growth rate, whereas η = 0 can be achieved either with *r*_1_ = *r*_2_ = 0, *r*_1_ *=* −*r*_2_, or −*r*_1_ = *r*_2_. The two approaches offer several advantages over ^14^C time-frequency analyses based on summed probability distribution (SPD). Bayesian growth models (*54*) account for sampling and measurement error, thereby overcoming key limitations of SPDs. Meanwhile, the ICAR component can benefit from partial pooling in the estimates of the key parameters of interest for each hexagonal cell. To explore the robustness of our change-point models, we considered three additional ICAR models for A and B with (i) no covariates, (ii) geographic distance from the caldera as linear predictors for *r*_2_ and η, and (iii) average depth of K-Ah tephra deposits as linear predictors for *r*_2_ and η. These alternative version were compared against the change-point version of our ICAR model using WAIC.

We fitted our models using the NIMBLE (*55*, *56*) and the nimbleCarbon (*54*, *57*) packages in R statistical computing language. Both models were fitted using four chains, each with 1,000,000 iterations (half discarded for burn-in) and sampled every 100 steps. Convergence was checked using the Gelman-Rubin statistic (*58*) and through visual inspections of the chains for the key parameters. Further details on the two models are provided in Supplementary Text.

## Supplementary Materials

### This PDF file includes:

Supplementary Text Equations S1 to S24 Figs. S1 to S8 Tables S1 to S4 References
