# Supplementary material for: Divergent demographic responses to the Kikai-Akahoya volcanic catastrophe in mid-Holocene Japan
Source: Sci Adv. 2026 Oct 14;12(42):eaeh7197. doi: 10.1126/sciadv.aeh7197 (PMC13665937; doi:10.1126/sciadv.aeh7197)
Supplement: Supplementary file 1 [file sciadv.aeh7197_sm.pdf]

Science Advances
AAAS

Supplementary Materials for

Divergent demographic responses to the Kikai-Akahoya volcanic catastrophe in mid-Holocene Japan

Enrico R. Crema *et al.*

Corresponding author: Enrico R. Crema, erc62@cam.ac.uk

Sci. Adv. **12**, eaeh7197 (2026)
DOI: 10.1126/sciadv.aeh7197

This PDF file includes:

Supplementary Text
Equations S1 to S24
Figs. S1 to S8
Tables S1 to S4
References

Supplementary Text

Extended Abstract in Japanese

「人口動態からみた鬼界アカホヤ巨大噴火が日本列島各地に与えた多様な影響」
クレーマ・E・R　茱畑光博　内山純蔵　ユーノ・A　リード・F　ジョーダン・P

本研究では、約7,300年前に九州南方海上で発生した鬼界アカホヤ巨大噴火が日本列島各地に与えた影響を、人口動態の分析を通して検討する。鬼界アカホヤ噴火は、完新世最大級の火山噴火として注目を集めてきた。考古学では従来、噴火が環境に与えた甚大な衝撃に対して、社会・文化の急激な崩壊を強調する立場が優勢だったが、近年では継続的なレジリエンスを強調する立場も現れ、活発な議論が行われてきた。しかし、鬼界アカホヤ噴火のような破局的災害の影響を、長期的視点から、日本列島規模の人口動態の軌跡の中に位置づける研究は少ないのが現状である。本研究では、鬼界アカホヤ噴火の前後の時期に注目し、放射性炭素年代値の時間頻度分布から、噴火が人口動態に与えた影響を推定するための新たなベイズ統計モデルを開発・適用した。具体的には、日本列島の地域ごとの噴火前（8000–7250 cal BP）および噴火後（7250–6500 cal BP）の人口増加率、ならびに年代値全体の比率の変化を、降灰堆積厚を予測変数とする「固有条件付き自己回帰モデル」（ICAR）によって推定する。その結果、鬼界アカホヤ噴火が、単純に激しい人口減少をもたらしたのではなく、各地域の条件に応じた複雑な影響を及ぼしたことが明らかになった。すなわち、噴火後の人口増加率は日本列島全体で概して低下し、とくに火山に近い地域（例：南九州）で顕著であった一方、他の近接地域（例：北九州、中国地方）では逆に人口が増加した証拠も示された。さらに、同時代における物質文化のうえで観察される地域的変化が、火山災害に対応した地域的移住を示唆している可能性についても検討する。先史・古代の大災害を人口動態の観点から分析することは、文化や社会が突発的・破局的ショックにどのように対処し、その対処がどのような条件に左右されるのかを理解するうえできわめて有用である。

Sample Preparation

Radiocarbon dates were obtained from version 1.2.0 of the curated English translation of the *Radiocarbon database of the National Museum of Japanese History* (*53*). We first considered ^{14}C dates meeting the following criteria:

1. Samples must be of terrestrial origin.
2. The cumulative calibrated probability of each sample between T_{start} and T_{end} must be larger than 0.5.
3. The sampling location must be within one of the four main islands of the Japanese archipelago (i.e., Hokkaido, Honshu, Shikoku, or Kyushu)

T_{start} and T_{end} were set to 8000 and 6500 BP to cover a 750-year span before and after 7250 BP, the approximate date of the K-Ah eruption. Additional sensitivity analyses were carried out using shorter (T_{start} = 7750 BP and T_{end} = 6750 BP, i.e. 500 years before and after the eruption) and longer (T_{start} = 8250 BP and T_{end} = 6250 BP, i.e. 1,000 years before and after the eruption) time spans (see Figure S1).
Given the often arbitrary nature of what constitutes an 'archaeological site', we first used DBSCAN *(59)* to generate clusters of archaeological sites using the geographic coordinates in the database, setting an *eps* distance of 100 meters. We then assigned a new sampling ID to each cluster, using the centroid of all original point locations as a new pair of coordinates. To account for inter-site variation in sampling intensity, we combined the *binPrep()* and the *thinDates()* function of the rcarbon R package *(60)*. The former was used to identify clusters from the same spatial sampling unit using a threshold of 100 years, while the latter was then used to randomly select one ^{14}C date among the observations with the lowest measurement error within each cluster. The final sample consisted of 540 ^{14}C dates from 324 sampling locations. Each sample was then assigned to one of the 61 hexagonal grids obtained by setting the *cellsize* argument on the *st_make_grid* function in the *sf* R package *(61,62)* set to 125 km. All analyses were performed using the IntCal20 calibration curve (*63*). The final sample size was n=540 in the 750yrs (i.e. T_{start} = 8000 and T_{end} = 6500), n=371 in the 500yrs (i.e. T_{start} = 7750 and T_{end} = 6750), and n=703 in the 1000 yrs (i.e. T_{start} = 8250 and T_{end} = 6250) time window setting.

Bayesian Modeling of ^{14}C dates

We fitted two custom Bayesian models (Model *a* and Model *b*) to examine spatial and temporal changes in the density of ^{14}C dates. Model *a* can be formalised with the following sets of equations:

$$\theta_{i,j} \sim DoubleExponentialGrowth(a=8{,}000, b=6{,}500, c=7.250, r_{1,j}, r_{2,j}) \quad [S1]$$

$$y_{i,j} \sim Normal(f(\theta_{i,j}), \sigma_i) \quad [S2]$$

$$r_{1,j} = s_{1,j} \quad [S3]$$

$$r_{2,j} = s_{2,j} + \beta_1 x_j + \beta_2 \max(0, x_j - \varkappa) \quad [S4]$$

$$s_{1,j} \sim \mathrm{ICAR}(\tau_1, \mathbf{W_j}) \quad [S5]$$

$$s_{2,j} \sim \mathrm{ICAR}(\tau_2, \mathbf{W_j}) \quad [S6]$$

$$\tau_1 = 1/\rho_1^2 \quad [S7]$$

$$\tau_2 = 1/\rho_2^2 \quad [S8]$$

$$\tau_1 \sim Uniform(0,100) \quad [S9]$$

$$\tau_2 \sim Uniform(0,100) \quad [S10]$$

$$\beta_1 \sim \mathrm{Normal}(0,0.1) \quad [S11]$$

$$\beta_2 \sim \text{Normal}(0,0.1) \quad [S12]$$

$$\varkappa \sim \text{TruncatedNormal}(0,3.21,2,0.1) \quad [S13]$$

Equation S1 describes the distribution of calendar dates $\theta_{i,j}$ from sample *i* in the hexagonal area *j* using a bounded double exponential growth model as described in *(54)* with a fixed change-point *c* corresponding to the estimated date (in BP) of the Kikai-Akahoya eruption (cf. main text, see also (*8*)). The model effectively describes the distribution of ^{14}C dates as a bounded Laplace distribution with rates $r_{1,j}$ and $r_{2,j}$. Equation S2 establishes the relationship between the true calendar date $\theta_{i,j}$ and the observed ^{14}C age through an error-in-variables model, where $f(\theta)$ is the corresponding ^{14}C age for the calendar date θ, and σ_i is the square root of the sum of the squares of the samples' ^{14}C age errors and the error on the calibration curve.

The two growth parameters are modelled with intrinsic conditional autoregressive (ICAR) models, implemented via the *dcar_normal* function in NIMBLE (equations S5 and S6). Here, the spatial parameters $s_{1,j}$ and $s_{2,j}$ were conditional on the ICAR model with the adjacency matrix $\mathbf{W_j}$ (effectively the immediate neighbourhood for each areal unit *j*) and the precision parameter τ, which defines the extent of the spatial autocorrelation. Growth rates before the eruption (i.e. $r_{1,j}$) were modelled entirely by ICAR (equations S3 and S5). In contrast, growth rates after the eruptions (i.e. $r_{2,j}$) were conditional on x_j, the base ten logarithm of the average thickness of the tephra deposit within each area *j*. More specifically, equation S4 is a change-point model where the effect of x_j on $r_{2,j}$ is determined by a slope of β_1 when $x_j \leq \varkappa$, and a slope of $\beta_1 + \beta_2$ when $x_j > \varkappa$. The prior for $\varkappa$ was weakly informative and centred around values (ca. 100 mm) above which the ecosystem impact is known to be disruptive at multi-centennial scales *(30,31)*.

Model *b* is defined by a similar set of equations:

$$\theta_{i,j} \sim \mathit{DoubleFlat}(a=8{,}000,\ b=6{,}500,\ c=7.250,\ \eta_j) \quad [S14]$$

$$y_{i,j} \sim \mathit{Normal}(f(\theta_{i,j}),\ \sigma_i) \quad [S15]$$

$$\eta_j = s_j + \beta_1 x_j + \beta_2 \max(0,\ x_j - \varkappa) \quad [S16]$$

$$s_j \sim \text{ICAR}(\tau,\mathbf{W_j}) \quad [S17]$$

$$\tau = 1/\rho^2 \quad [S18]$$

$$\tau \sim \mathit{Uniform}(0,100) \quad [S19]$$

$$\beta_1 \sim \text{Normal}(0,0.01) \quad [S20]$$

$$\beta_2 \sim \text{Normal}(0,0.01) \quad [S21]$$

$$\varkappa \sim \text{TruncatedNormal}(0,3.21,2,0.1) \quad [S22]$$

The key difference is that $\theta_{i,j}$ is now modelled by the bounded distribution *DoubleFlat,* which simply defines the probability of a given sample being from the calendar year t as

$$P(\theta = t \mid a,b,c,\eta,t \geq c) = (1- \text{logit}^{-1}(\eta)) / a-c \quad [S23]$$

$$P(\theta = t \mid a,b,c,\eta,t < c) = \text{logit}^{-1}(\eta) / c-b \quad [S24]$$

Given a-c and c-b are the same in our case (see equation S14), the parameter η will effectively lead to a higher probability of θ being dated (in BP) between c and b when positive, and a lower probability when negative. When $\eta = 0$, equation S14 effectively models the distribution of θ to be uniform between a and b.

Equation S14 has a single free parameter (the log-odds of the sample being dated between c and b), which is conditional to a spatially varying baseline s_j and the effect of the average thickness of the tephra deposit within each area j (equation S16).

Three variants for the two models have also been considered with the following modifications:

- Intercept-only models without any covariates, i.e. $r_{2,j} = s_{2,j}$ for model A and $\eta_j = s_j$ for model B (models A_0 and B_0)
- Linear models where equations S4 and S16 are replaced by a simple regression $r_{2,j} = s_{2,j} + \beta_1 x_j$ and $\eta_j = s_j + \beta_1 x_j$, x_j equations, using the average geographic distance from the caldera (models A_{dist} and B_{dist}) and the average thickness of the tephra deposit (models A_{tephra} and B_{tephra}) as predictors.

Table S1 summarises the WAIC comparison between the main models and these variants.

Implementation in NIMBLE

The two models were designed and fitted using the NIMBLE *(55,56)* probabilistic programming language in R, with additional functions from the *nimbleCarbon* package *(54,57)*. Both models were fitted using four chains with the following MCMC settings: 1,000,000 iterations, 500,000 burn-in steps, and 100 thinning steps. Effective sample size and Gelman-Rubin statistics (see tables S3 and S4 below) were computed using the *coda* R package *(64)*.

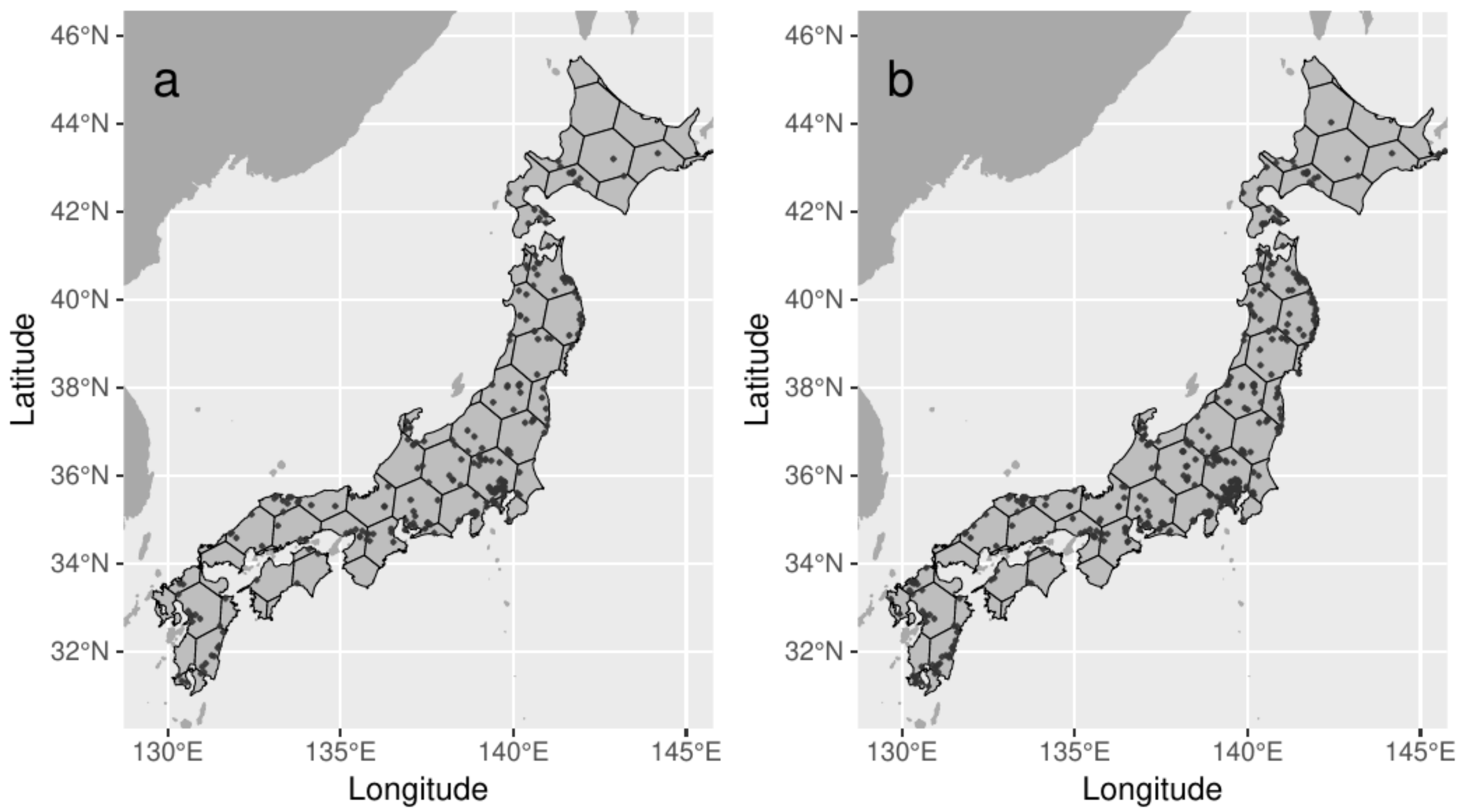

Fig. S1. Sample Distribution Map

Distribution of sampling locations for the 1,000–year (7750–6750 BP) and 2,000–year (8250–6250 BP) time windows of analyses.

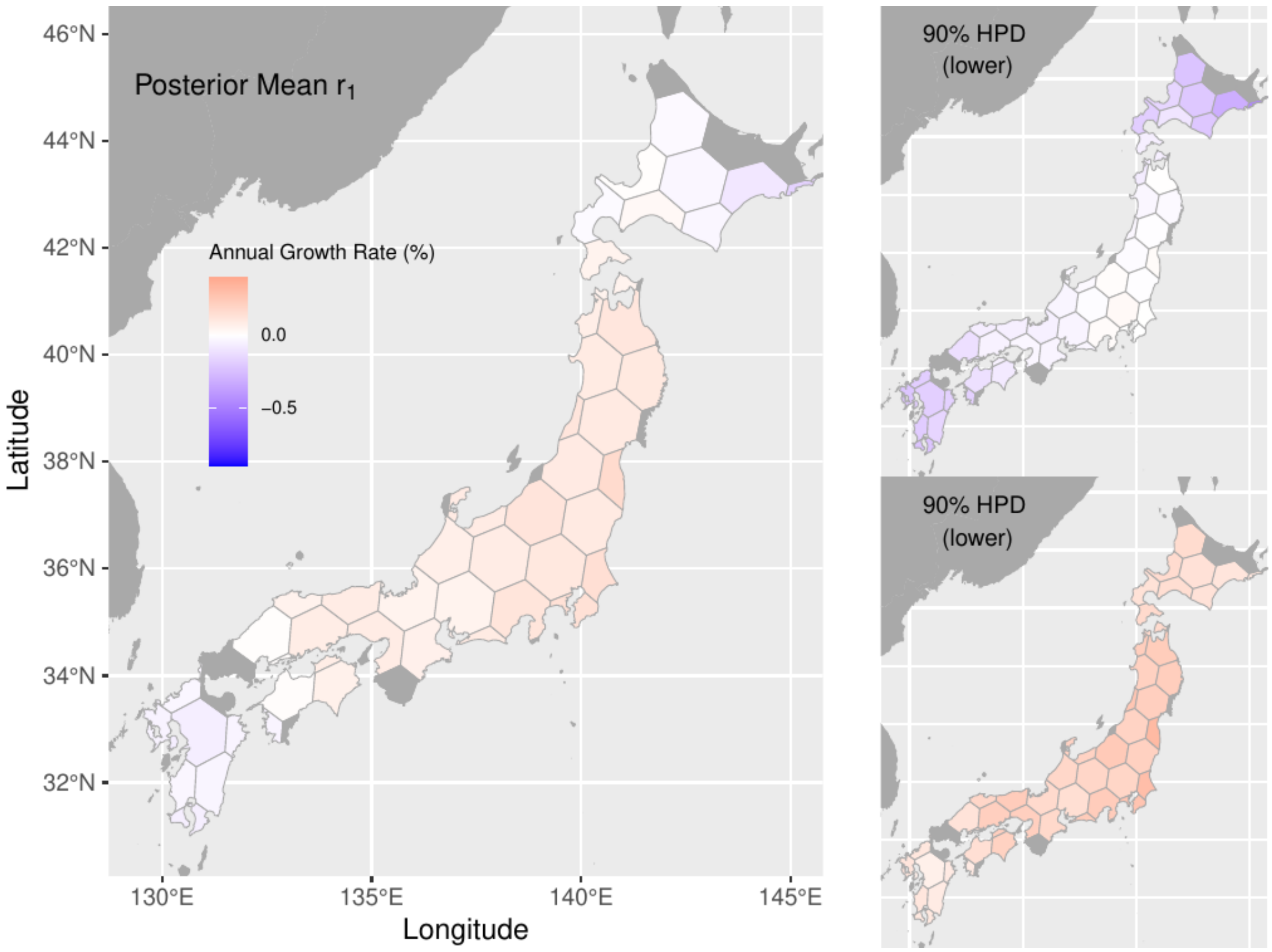

Fig. S2. Posterior of r_1

Posterior mean and the lower/higher 90% highest posterior density interval for r_1 in model *A*.

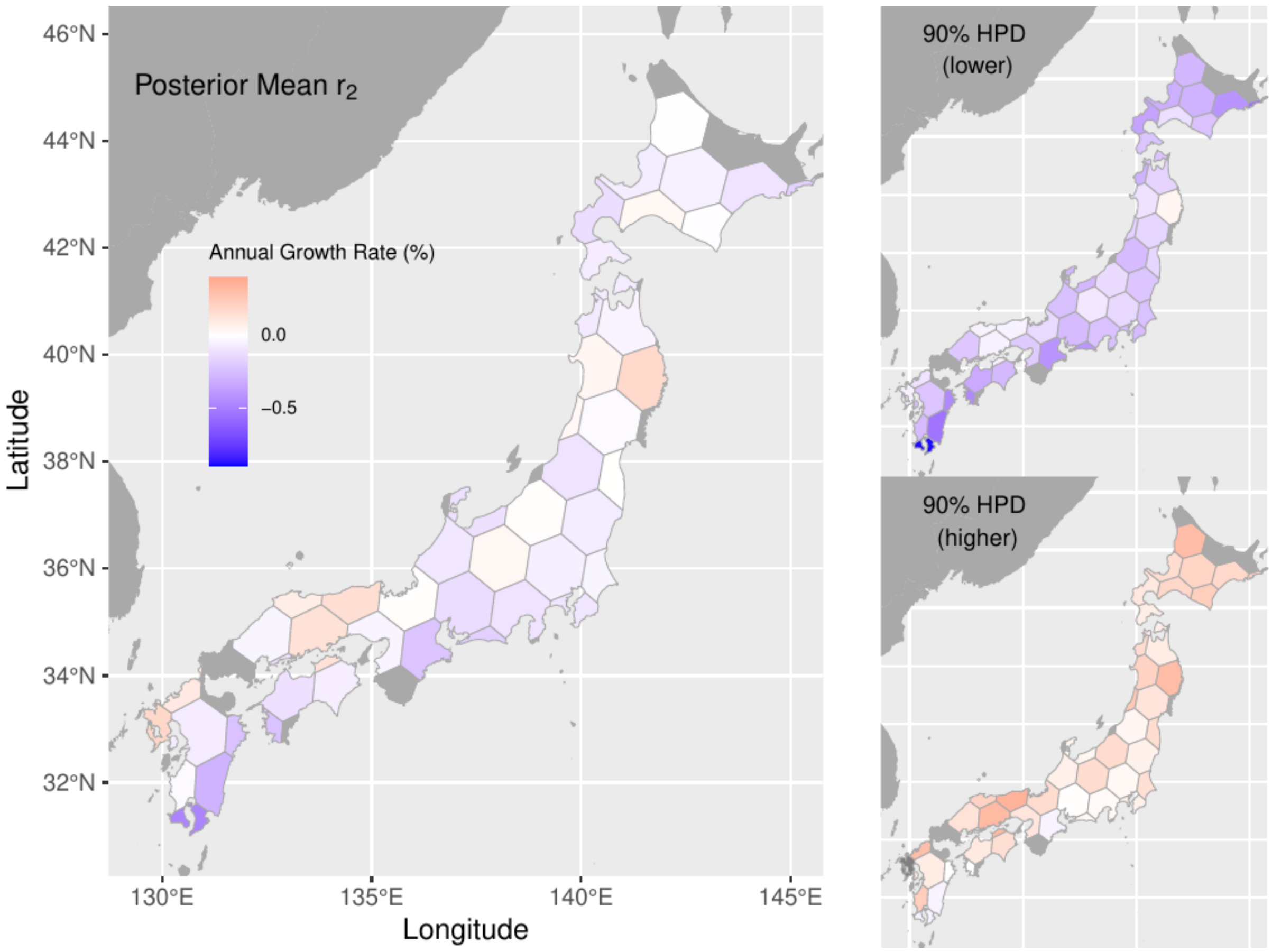

Fig. S3. Posterior of r_2

Posterior mean and the lower/higher 90% highest posterior density interval for r_2 in model *a*.

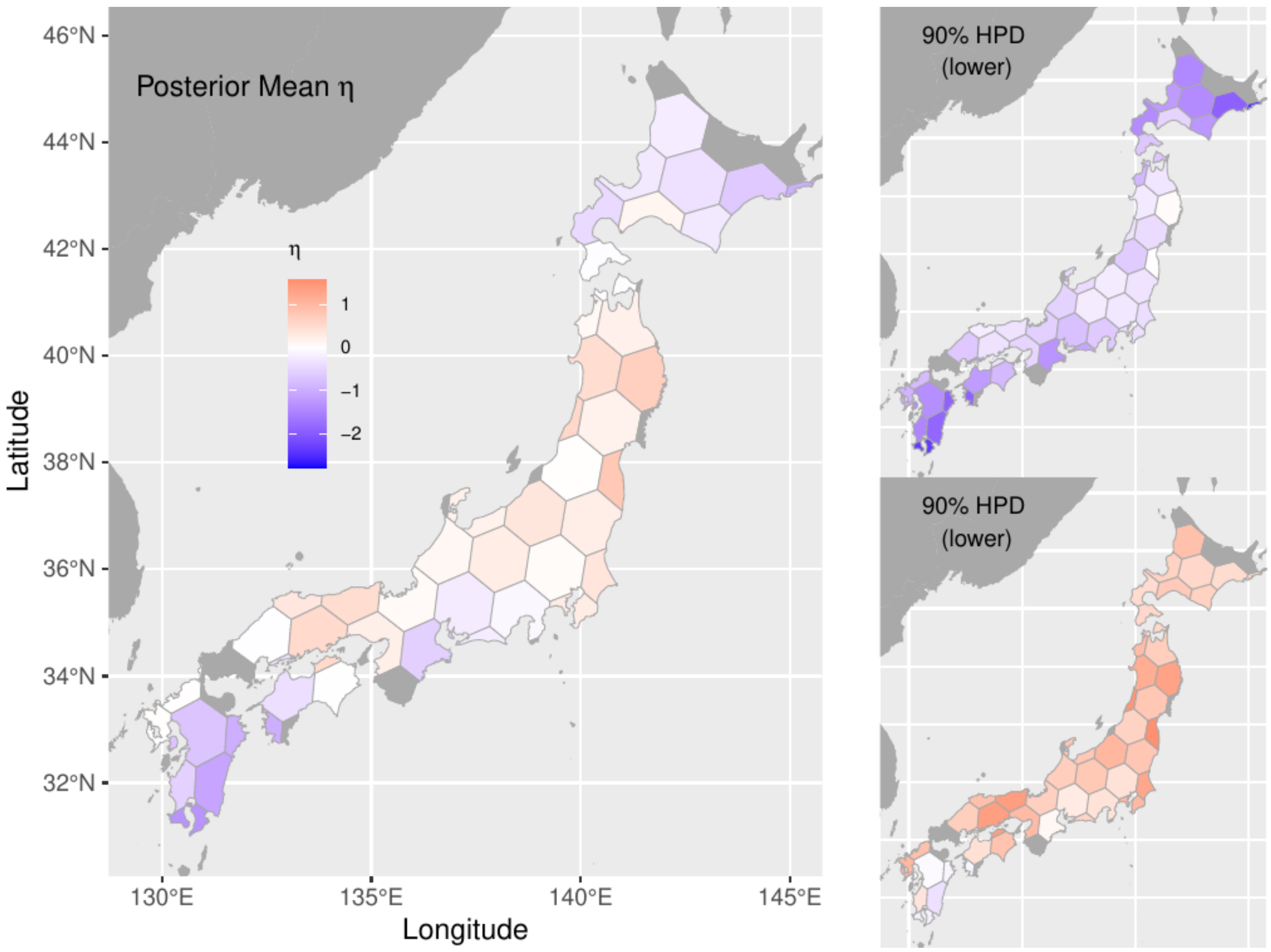

Fig. S4. Posterior of η

Posterior mean and the lower/higher 90% highest posterior density interval for η in model *b*.

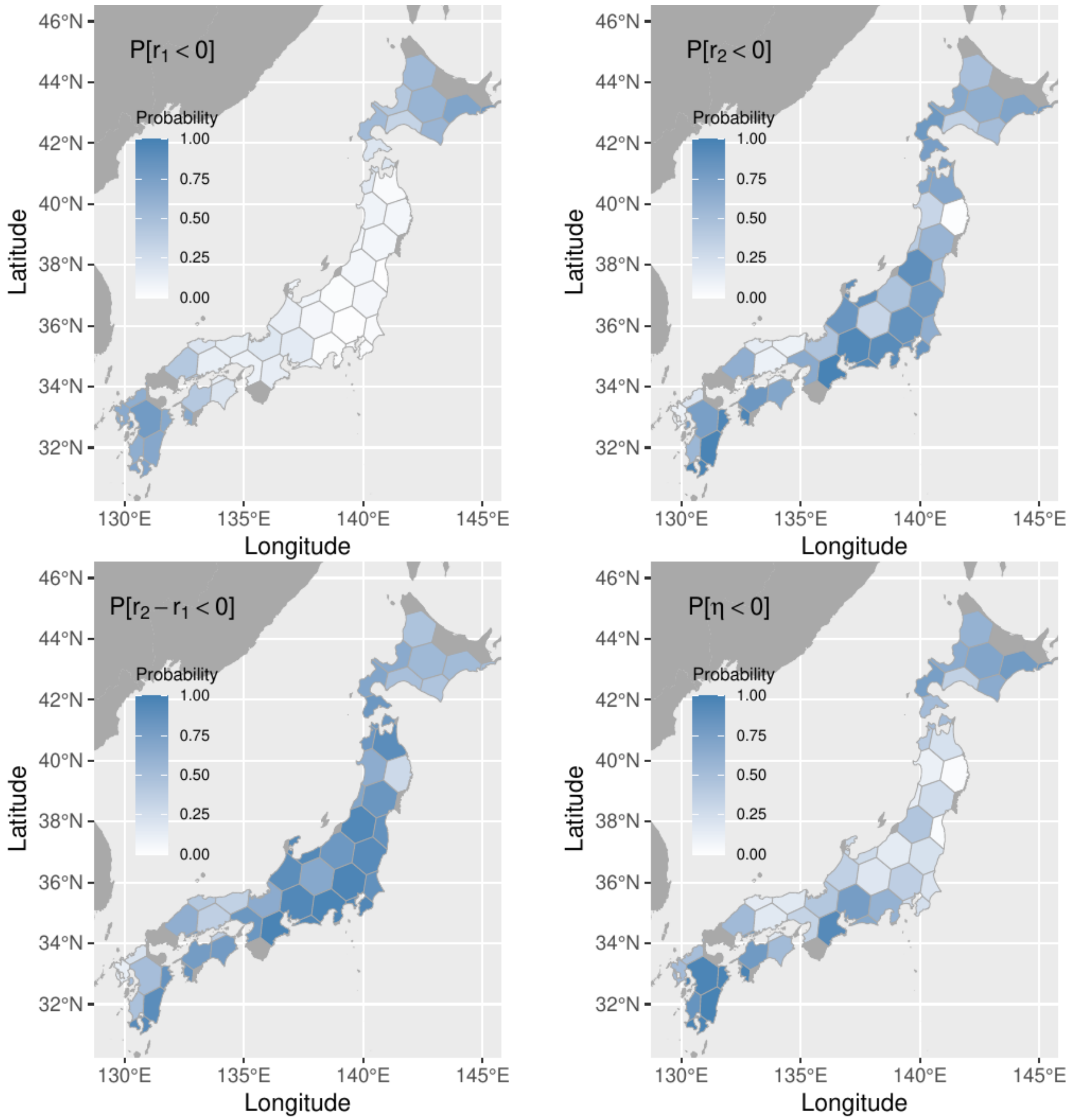

Fig. S5. Cumulative posterior probabilities of key parameters

Cumulative posterior probability for $r_1 < 0$ (top left) $r_2 < 0$ (top right), $r_2 - r_1 < 0$, and $\eta < 0$.

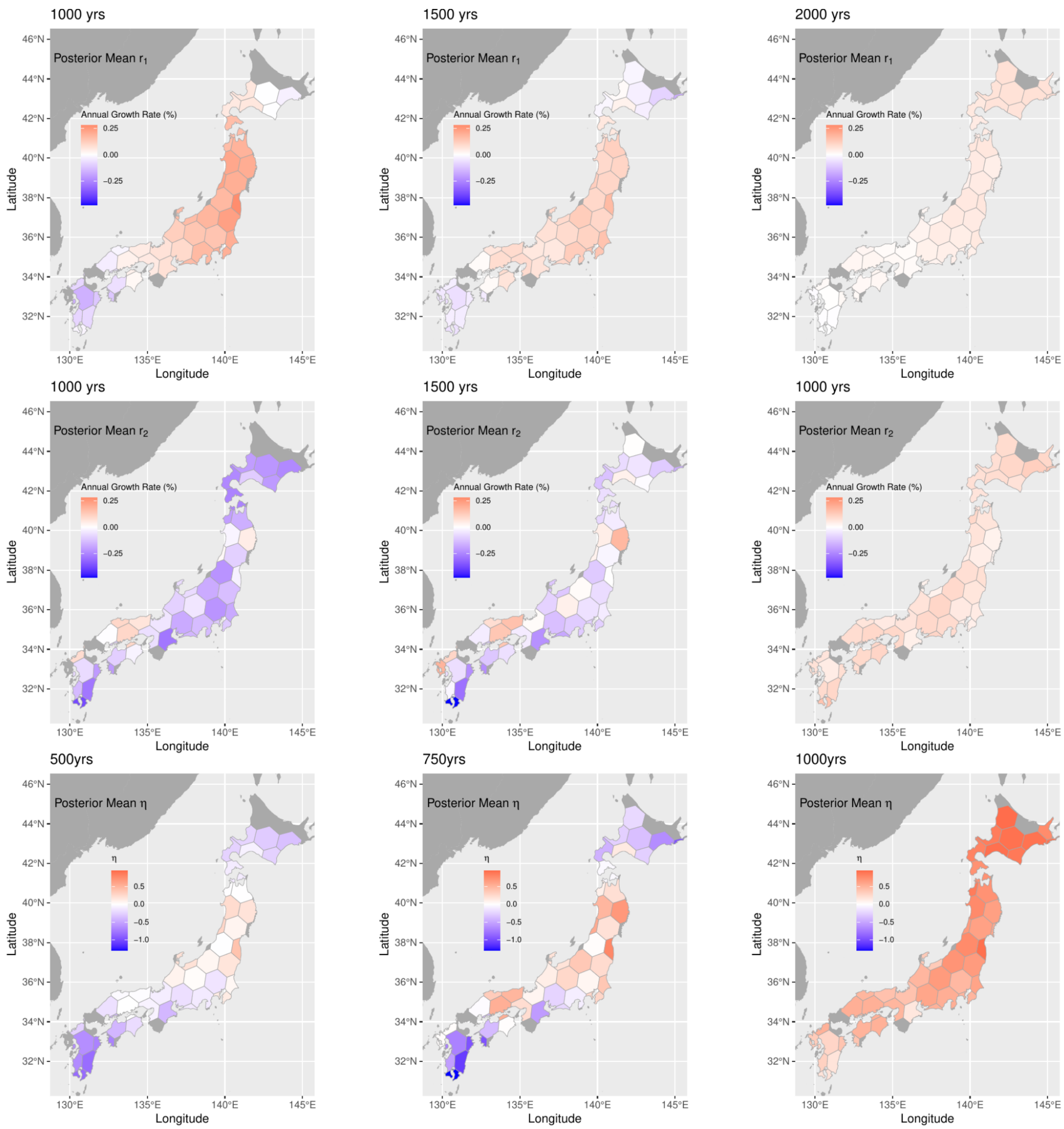

Fig. S6. Sensitivity analyses for model parameters under different time windows.

Posterior means for r_1, r_2, and η for the 1,000–year (7750–6750 BP), 1,500 –year (8000–6500 BP), and 2,000–year (8250– 6250 BP) time windows

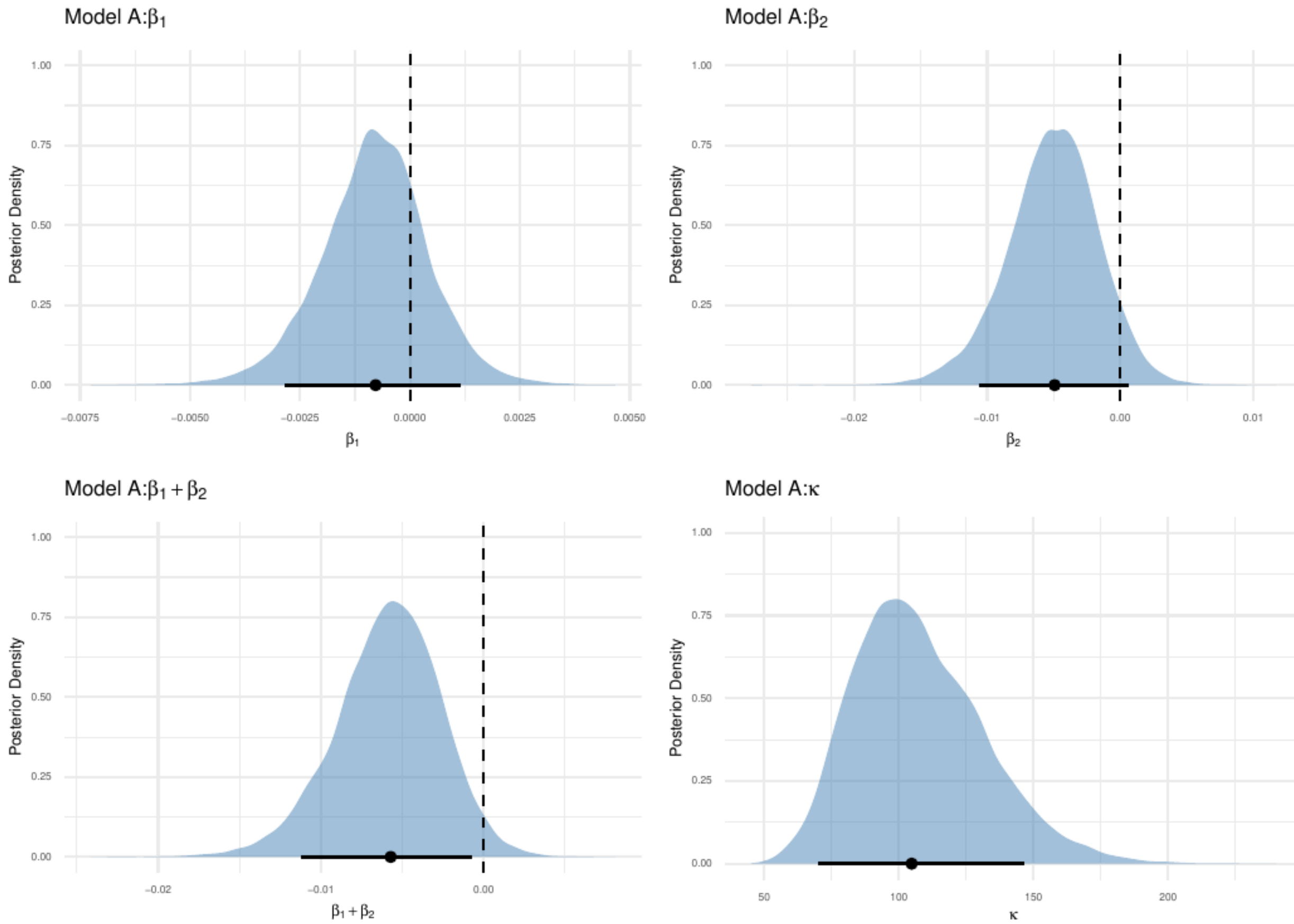

Fig. S7 Posterior of covariate parameters for model A

Posterior distributions of model A's β_1, β_2, $\beta_1 + \beta_2$, and $\varkappa$.

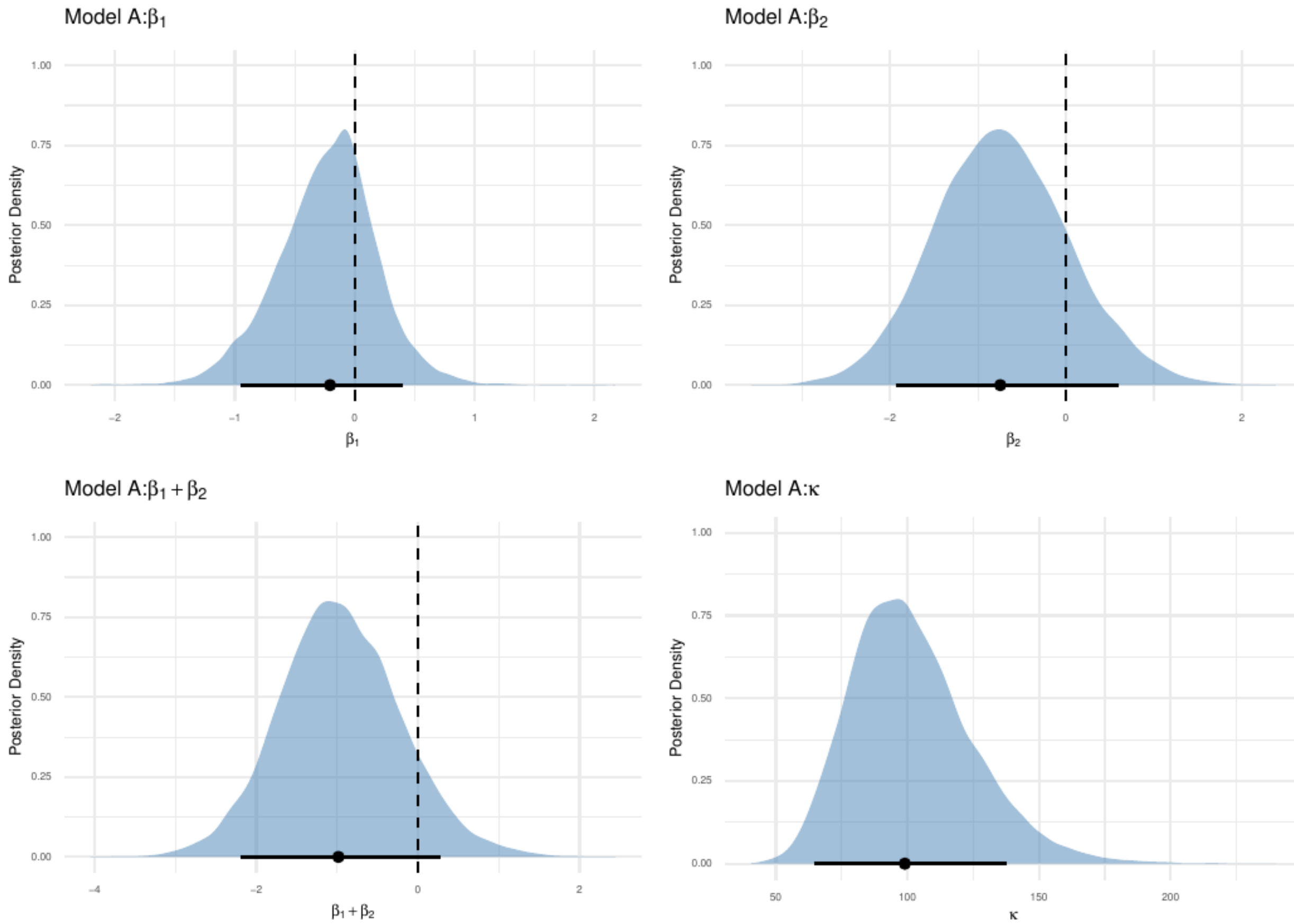

Fig. S8 Posterior of covariate parameters for model B
Posterior distributions of model B's β_1, β_2, $\beta_1 + \beta_2$, and $\varkappa$.

Table S1.
WAIC model comparison for model A. ΔWAIC[1] refers to the difference in the WAIC values against the best-performing model, whilst ΔWAIC[1] refers to the difference against the main model.

Model	WAIC	ΔWAIC[1]	ΔWAIC[2]	WAIC weight
Main Model (A)	6353.263	0.541	0	0.233
A_0	6353.507	0.785	0.243	0.206
A_{dist}	6352.722	0	-0.541	0.305
A_{tephra}	6353.076	0.354	-0.187	0.256

Table S2.
WAIC model comparison for model B. ΔWAIC[1] refers to the difference in the WAIC values against the best-performing model, whilst ΔWAIC[1] refers to the difference against the main model.

Model	WAIC	ΔWAIC[1]	ΔWAIC[2]	WAIC weight
Main Model (B)	6352.052	1.469	0	0.223
B_0	6350.583	0.000	-1.469	0.465
B_{dist}	6353.020	2.437	0.968	0.137
B_{tephra}	6352.541	1.958	0.489	0.175

Table S3.

Posterior mean, highest posterior density interval, and convergence diagnostics for model *A*.

Parameter	Posterior Mean	90% HPDI (low)	90% HPDI (high)	Effective Sample Size	R-hat
beta1	-0.000814	-0.002828	0.001184	1116.0	1.00
beta2	-0.005069	-0.010647	0.000613	9365.4	1.00
kappa	2.020602	1.867285	2.184028	19755.3	1.00
s1[1]	-0.000458	-0.002196	0.001217	10345.2	1.00
s1[2]	-0.000340	-0.001942	0.001173	11325.3	1.00
s1[3]	-0.000349	-0.001895	0.001145	11602.7	1.00
s1[4]	-0.000607	-0.002250	0.001109	9339.9	1.00
s1[5]	-0.000644	-0.001850	0.000725	5837.2	1.00
s1[6]	-0.000151	-0.002048	0.001578	14884.6	1.00
s1[7]	-0.000391	-0.001604	0.000948	8504.3	1.00
s1[8]	-0.000221	-0.001660	0.001170	10326.3	1.00
s1[9]	-0.000404	-0.001752	0.000969	8546.7	1.00

s1[10]	0.000109	-0.001225	0.001328	14238.4	1.00
s1[11]	0.000126	-0.001189	0.001509	13666.9	1.00
s1[12]	0.000627	-0.000670	0.001925	17565.0	1.00
s1[13]	0.000130	-0.001749	0.001835	19212.0	1.00
s1[14]	0.000863	-0.000486	0.002256	14316.6	1.00
s1[15]	0.000677	-0.000818	0.002131	16448.5	1.00
s1[16]	0.000904	-0.000620	0.002378	16253.5	1.00
s1[17]	0.000890	-0.000344	0.002238	14739.0	1.00
s1[18]	0.000763	-0.001082	0.002468	18684.3	1.00
s1[19]	0.000587	-0.000579	0.001740	18893.0	1.00
s1[20]	0.000850	-0.001112	0.002961	19183.3	1.00
s1[21]	0.000699	-0.000420	0.001799	19443.2	1.00
s1[22]	0.000748	-0.000384	0.001891	18910.0	1.00
s1[23]	0.000572	-0.000418	0.001517	17292.3	1.00
s1[24]	0.000931	-0.000229	0.002105	18487.7	1.00
s1[25]	0.000966	-0.000355	0.002409	19176.6	1.00
s1[26]	0.000897	-0.000120	0.001910	17991.1	1.00

s1[27]	0.001085	-0.000391	0.002657	18547.2	1.00
s1[28]	0.000406	-0.001545	0.002091	16691.3	1.00
s1[29]	-0.000045	-0.002421	0.002126	14262.9	1.00
s1[30]	0.001246	0.000175	0.002378	13029.8	1.00
s1[31]	0.001255	0.000150	0.002480	13018.3	1.00
s1[32]	0.001211	-0.000289	0.002671	15483.1	1.00
s1[33]	0.000815	-0.000716	0.002307	18512.7	1.00
s1[34]	-0.000186	-0.001884	0.001375	9006.4	1.00
s1[35]	0.001215	-0.000457	0.003021	16801.3	1.00
s1[36]	0.001104	0.000265	0.001941	13265.6	1.00
s1[37]	0.000973	-0.000169	0.002077	18598.9	1.00
s1[38]	0.000951	-0.000344	0.002155	17566.8	1.00
s1[39]	0.000579	-0.000621	0.001738	15662.3	1.00
s1[40]	0.000113	-0.001555	0.001676	13120.0	1.00
s1[41]	-0.000296	-0.003238	0.002297	14620.5	1.00
s1[42]	0.001419	0.000013	0.002759	11160.3	1.00
s1[43]	0.000995	-0.000069	0.002152	17224.7	1.00

s1[44]	0.001013	-0.000089	0.002250	18403.8	1.00
s1[45]	0.001177	0.000044	0.002373	13221.7	1.00
s1[46]	0.000321	-0.001004	0.001573	13073.1	1.00
s1[47]	-0.000210	-0.002283	0.001667	11106.9	1.00
s1[48]	0.001487	0.000022	0.003047	12667.8	1.00
s1[49]	0.001698	0.000196	0.003248	10036.9	1.00
s1[50]	0.001061	-0.000217	0.002294	15708.7	1.00
s1[51]	0.000543	-0.000928	0.001996	18062.0	1.00
s1[52]	-0.000330	-0.002122	0.001394	8474.5	1.00
s1[53]	-0.000385	-0.002962	0.001973	12186.8	1.00
s1[54]	0.001207	-0.000347	0.003048	16910.0	1.00
s1[55]	0.000925	-0.000852	0.002649	18642.9	1.00
s1[56]	-0.000334	-0.002149	0.001358	8270.9	1.00
s1[57]	-0.000652	-0.002835	0.001450	7727.0	1.00
s1[58]	-0.000900	-0.003145	0.001205	6979.5	1.00
s1[59]	-0.001409	-0.004119	0.001246	6094.9	1.00
s1[60]	-0.001684	-0.004629	0.001170	5660.6	1.00

s1[61]	-0.001539	-0.004800	0.001484	7442.6	1.00
s2[1]	0.004647	-0.000235	0.009900	1395.3	1.00
s2[2]	0.004171	-0.001286	0.009563	1538.0	1.00
s2[3]	0.003450	-0.001327	0.008134	1332.0	1.00
s2[4]	0.003876	-0.002183	0.010279	1975.2	1.00
s2[5]	0.002829	-0.001886	0.007571	1328.2	1.00
s2[6]	0.002821	-0.002365	0.008005	1638.9	1.00
s2[7]	0.003436	-0.002046	0.008857	1590.1	1.00
s2[8]	0.002675	-0.002003	0.007776	1395.1	1.00
s2[9]	0.002101	-0.002634	0.007327	1482.3	1.00
s2[10]	0.002341	-0.002208	0.006886	1286.1	1.00
s2[11]	0.002529	-0.002103	0.007555	1389.5	1.00
s2[12]	0.002748	-0.001444	0.007097	1179.4	1.00
s2[13]	0.002552	-0.002599	0.007993	1640.9	1.00
s2[14]	0.004438	-0.000132	0.009234	1281.1	1.00
s2[15]	0.003080	-0.001668	0.007977	1346.5	1.00
s2[16]	0.003778	-0.000717	0.008589	1325.3	1.00

s2[17]	0.002748	-0.001590	0.007500	1274.8	1.00
s2[18]	0.002204	-0.003040	0.007255	1585.1	1.00
s2[19]	0.002554	-0.001653	0.007245	1239.4	1.00
s2[20]	0.000597	-0.004411	0.005583	2031.9	1.00
s2[21]	0.000808	-0.003673	0.005355	1349.5	1.00
s2[22]	0.000817	-0.003355	0.005039	1247.7	1.00
s2[23]	0.000975	-0.003278	0.005222	1215.4	1.00
s2[24]	0.000354	-0.003581	0.004334	1293.5	1.00
s2[25]	0.000940	-0.003828	0.005401	1387.2	1.00
s2[26]	0.002106	-0.001807	0.006414	1178.3	1.00
s2[27]	0.000733	-0.003052	0.004555	1889.7	1.00
s2[28]	-0.000909	-0.004093	0.001865	17603.1	1.00
s2[29]	-0.000971	-0.004717	0.002709	17314.4	1.00
s2[30]	0.000928	-0.003169	0.005137	1174.3	1.00
s2[31]	0.001618	-0.002178	0.005574	1249.7	1.00
s2[32]	0.000788	-0.001964	0.003424	2592.0	1.00
s2[33]	-0.000796	-0.002972	0.001654	17977.8	1.00

s2[34]	-0.001251	-0.003562	0.001106	14417.7	1.00
s2[35]	0.000899	-0.003816	0.005909	1596.2	1.00
s2[36]	0.001073	-0.002932	0.005064	1150.8	1.00
s2[37]	0.000152	-0.003108	0.003232	1443.5	1.00
s2[38]	0.000428	-0.001125	0.002056	11906.6	1.00
s2[39]	-0.000671	-0.002224	0.000807	16292.8	1.00
s2[40]	-0.000721	-0.003096	0.001607	16063.9	1.00
s2[41]	-0.000082	-0.004420	0.004294	19495.3	1.00
s2[42]	0.000820	-0.003416	0.005106	1269.8	1.00
s2[43]	0.000751	-0.003021	0.004506	1277.1	1.00
s2[44]	0.000353	-0.001688	0.002401	2161.8	1.00
s2[45]	-0.000408	-0.001630	0.000884	17145.8	1.00
s2[46]	0.000354	-0.001205	0.001907	17658.3	1.00
s2[47]	0.000033	-0.002792	0.003135	18447.4	1.00
s2[48]	0.001173	-0.003070	0.005431	1379.0	1.00
s2[49]	0.001187	-0.001816	0.004318	1438.5	1.00
s2[50]	0.001763	0.000415	0.003148	10788.5	1.00

s2[51]	-0.000083	-0.002302	0.002217	19398.6	1.00
s2[52]	-0.000543	-0.002944	0.001976	15828.8	1.00
s2[53]	-0.000238	-0.003945	0.003736	19045.7	1.00
s2[54]	0.000531	-0.002364	0.003421	4519.2	1.00
s2[55]	0.000411	-0.002344	0.003160	20216.5	1.00
s2[56]	-0.000080	-0.002333	0.002269	16814.6	1.00
s2[57]	-0.000642	-0.003720	0.002467	16566.6	1.00
s2[58]	-0.001058	-0.004110	0.001791	14528.1	1.00
s2[59]	-0.001427	-0.005032	0.002176	13799.7	1.00
s2[60]	-0.001710	-0.005621	0.002101	13591.7	1.00
s2[61]	-0.001562	-0.006446	0.002828	16751.7	1.00
sigma1	0.001407	0.000306	0.002455	3946.9	1.00
sigma2	0.002562	0.001177	0.003913	6647.2	1.00

Table S4.
Posterior mean, highest posterior density interval, and convergence diagnostics for model *B*.

Parameter	Posterior Mean	90% HPDI (low)	90% HPDI (high)	Effective Sample Size	R-hat
beta1	-0.2318	-0.9342	0.4199	1612.7	1.00
beta2	-0.7281	-1.9362	0.5927	11135.7	1.00
eta[1]	0.7052	-1.0378	2.5572	2162.7	1.00
eta[2]	0.3978	-1.4740	2.3662	2266.4	1.00
eta[3]	0.6468	-0.9829	2.4214	2051.3	1.00
eta[4]	0.3442	-1.7913	2.5088	2385.0	1.00
eta[5]	0.0991	-1.5230	1.8244	2074.5	1.01
eta[6]	0.5661	-1.1705	2.5727	2501.9	1.00
eta[7]	0.1858	-1.7730	2.0397	2282.4	1.00
eta[8]	0.4048	-1.2483	2.1968	2171.6	1.01
eta[9]	-0.0138	-1.8356	1.7597	2383.3	1.01
eta[10]	0.6517	-0.8901	2.2947	1895.2	1.00
eta[11]	0.4500	-1.2337	2.1856	2061.7	1.00
eta[12]	0.8760	-0.5437	2.4440	1759.6	1.00
eta[13]	0.4302	-1.3960	2.4123	2554.1	1.00

eta[14]	1.2653	-0.3927	3.0075	1816.0	1.00
eta[15]	0.8483	-0.8101	2.6011	2114.3	1.00
eta[16]	1.0876	-0.5898	2.7892	1945.9	1.00
eta[17]	0.9750	-0.5706	2.7148	1924.6	1.00
eta[18]	0.6621	-1.0663	2.5877	2515.0	1.00
eta[19]	0.6997	-0.7460	2.3363	1836.6	1.00
eta[20]	0.6176	-1.1490	2.5014	3085.8	1.00
eta[21]	0.1742	-1.3822	1.7937	1996.2	1.01
eta[22]	0.6060	-0.8123	2.1041	1878.3	1.00
eta[23]	0.3596	-1.0777	1.8337	1794.1	1.00
eta[24]	0.6165	-0.6525	2.0772	1883.3	1.00
eta[25]	0.4654	-1.1540	2.0829	2150.1	1.00
eta[26]	0.7562	-0.5680	2.2707	1774.8	1.00
eta[27]	0.6230	-0.6964	2.0748	2884.8	1.00
eta[28]	-0.1278	-1.2840	1.0306	16329.8	1.00
eta[29]	-0.3552	-1.8415	1.0115	15149.8	1.00
eta[30]	0.4488	-0.9613	1.9341	1867.0	1.00

eta[31]	0.8016	-0.4607	2.2227	1953.3	1.00
eta[32]	0.7033	-0.3690	1.8298	3491.2	1.00
eta[33]	0.1039	-0.8498	0.9621	19769.0	1.00
eta[34]	-0.4351	-1.3766	0.5133	8226.8	1.00
eta[35]	0.5631	-1.0915	2.3584	2585.2	1.00
eta[36]	0.5431	-0.7544	1.9426	1692.2	1.00
eta[37]	0.3729	-0.7261	1.4742	2255.8	1.00
eta[38]	0.4902	-0.2436	1.1852	8265.6	1.00
eta[39]	-0.0355	-0.6606	0.5638	12994.7	1.00
eta[40]	-0.2641	-1.2025	0.6611	11773.5	1.00
eta[41]	-0.2642	-2.0198	1.4144	18482.1	1.00
eta[42]	0.7759	-0.6927	2.3307	1871.5	1.00
eta[43]	0.6607	-0.5806	2.0168	1942.2	1.00
eta[44]	0.3618	-0.4178	1.1479	3641.0	1.00
eta[45]	0.2167	-0.2989	0.7151	15876.0	1.00
eta[46]	0.1456	-0.5085	0.7632	18255.8	1.00
eta[47]	-0.2112	-1.4292	0.9026	16430.6	1.00

eta[48]	0.8296	-0.5976	2.4626	2141.3	1.00
eta[49]	1.1187	-0.0833	2.3418	2186.2	1.00
eta[50]	0.6848	0.0527	1.3311	6006.6	1.00
eta[51]	0.0735	-0.7971	0.9682	18560.9	1.00
eta[52]	-0.3531	-1.3938	0.5617	11039.0	1.00
eta[53]	-0.3143	-1.8373	1.2075	17025.5	1.00
eta[54]	0.5060	-0.6069	1.6283	6206.9	1.00
eta[55]	0.3269	-0.7126	1.4426	19284.8	1.00
eta[56]	-0.2642	-1.2444	0.6558	12440.9	1.00
eta[57]	-0.4699	-1.6893	0.7699	10970.2	1.00
eta[58]	-0.6382	-1.9026	0.5082	7895.9	1.00
eta[59]	-0.8199	-2.3173	0.6064	7596.3	1.00
eta[60]	-0.9643	-2.5021	0.5789	6890.2	1.00
eta[61]	-0.8842	-2.7789	0.8456	9892.5	1.00
kappa	1.9961	1.8394	2.1602	19392.9	1.00
sigma1	0.9599	0.2106	1.6584	3060.0	1.01

REFERENCES

1. S. Shimizu, R. Nakaoka, N. Seama, K. Suzuki-Kamata, K. Kaneko, K. Kiyosugi, H. Iwamaru, M. Sano, T. Matsuno, H. Sugioka, Y. Tatsumi, Submarine pyroclastic deposits from 7.3 ka caldera-forming Kikai-Akahoya eruption. *J. Volcanol. Geotherm. Res.* **448**, 108017 (2024).

2. P. D. Sheets, J. Cooper, “Learning to live with the dangers of sudden environmental change” in *Surviving Sudden Environmental Change*, J. Cooper, P. D. Sheets, Eds. (Univ. Press of Colorado, 2012), pp. 1–18.

3. F. Riede, P. D. Sheets, “Introduction. Framing catastrophes archaeologically” in *Going Forward by Looking Back: Archaeological Perspectives on Socio-Ecological Crisis, Response, and Collapse*, F. Riede, P. D. Sheets, Eds. (Berghahn Books, 2020), pp. 1–14.

4. G. D. Middleton, Nothing lasts forever: Environmental discourses on the collapse of past societies. *J. Archaeol. Res.* **20**, 257–307 (2012).

5. J. Habu, *Ancient Jomon of Japan* (Univ. of Cambridge Press, 2004).

6. C. G. Newhall, S. Self, The volcanic explosivity index (VEI) an estimate of explosive magnitude for historical volcanism. *J. Geophys. Res.* **87**, 1231–1238 (1982).

7. P. G. Albert, V. C. Smith, T. Suzuki, D. McLean, E. L. Tomlinson, Y. Miyabuchi, I. Kitaba, D. F. Mark, H. Moriwaki, T. Nakagawa, Geochemical characterisation of the Late Quaternary widespread Japanese tephrostratigraphic markers and correlations to the Lake Suigetsu sedimentary archive (SG06 core). *Quat. Geochronol.* **52**, 103–131 (2019).

8. V. C. Smith, R. A. Staff, S. P. E. Blockley, C. Bronk Ramsey, T. Nakagawa, D. F. Mark, K. Takemura, T. Danhara, Identification and correlation of visible tephras in the Lake Suigetsu SG06 sedimentary archive, Japan: Chronostratigraphic markers for synchronising of east Asian/west Pacific palaeoclimatic records across the last 150 ka. *Quat. Sci. Rev.* **67**, 121–137 (2013).

9. Y. Haruta, F. Maeno, Y. J. Suzuki, Reconstruction of the plinian phase of the 7.3 ka "Akahoya" caldera-forming eruption at the Kikai caldera, Japan. *J. Volcanol. Geotherm. Res.* **472**, 108541 (2026).

10. T. Kobayashi, Kikai Karudera no 7.3 cal ka BP funka ni kanren suru Akahoya tsunami no zentaizō [Overall view of the Akahoya tsunami after the 7.3 cal ka BP Kikai Caldera-forming eruption]. *Bull. Volcanol. Soc. Jpn.* **67**, 335–350 (2022).

11. C. Y. Di Prinzio, B. Penaluna, M. G. Grech, L. M. Manzo, M. L. Miserendino, R. Casaux, Impact of Chaitén Volcano ashfall on native and exotic fish recovery, recolonization, and abundance. *Sci. Total Environ.* **752**, 141864 (2021).

12. K. M. Bisson, S. Gassó, N. Mahowald, S. Wagner, B. Koffman, S. A. Carn, S. Deutsch, E. Gazel, S. Kramer, N. Krotkov, C. Mitchell, M. E. Pritchard, K. Stamieszkin, C. Wilson, Observing ocean ecosystem responses to volcanic ash. *Remote Sens. Environ.* **296**, 113749 (2023).

13. G. L. Barnes, "Introduction to TephroArchaeology" in *TephroArchaeology in the North Pacific*, G. L. Barnes, T. Soda, Eds. (Archaeopress, 2019), pp. 1–13.

14. S. Azharuddin, K. Omine, K. Masaka, R. Asami, M. A. Lone, Y.-C. Chou, C.-C. Shen, R. Uemura, Multidecadal hydroclimate responses to volcanic forcing in the Mid-Holocene. *Commun. Earth Environ.* **6**, 101 (2025).

15. R. Pearson, Jomon hot spot: Increasing sedentism in south-western Japan in the Incipient Jomon (14,000–9250 cal.bc) and Earliest Jomon (9250–5300 cal.bc) periods. *World Archaeol.* **38**, 239–258 (2006).

16. K. Shintō, "Kazanbai karamita Minami Kyushu Jomon Sō-Zen ki doki no Yōsō [Typological sequences of pottery designs in Southern Kyushu from the Initial to Early phases as seen from the perspective of volcanic tephra]" in *Kagamiyama Takeshi Sensei Koki Kinen Kobunka Ronkō [Festschrift of Ancient Culture Theses for Professor Takeshi Kagamiyama on His 70th Birthday]* (Association of Publishing the Festschrift for Professor Takashi Kagamiyama, 1980), pp. 11–23.

17. Y. Kizaki, “Kumamoto ken daimaru fujigasako iseki no senokan shiki doki nitsuite [Senokan style pottery at the Daimaru Fujigasako Iseki, Kumamoto Prefecture]” in *Senokan Shiki Doki: Chimei Hyō, Takuei, Ronkō Hen [Senokan Style Pottery: Collection of Site Names, Rubbings and Discussions]* (Jōmon Kenkyukai, 1985), pp. 158–169.

18. M. Kuwahata, *Chō Kyodaifunka Ga Jinrui Ni Ataeta Eikyō [Impact of the Kikai-Akahoya Super-Eruption on Humans]* (Yūzankaku, 2016).

19. M. Kuwahata, Kyushu ni okeru kikai akahoya funka zengo no Jōmon iseki no dōtai [The impact of the Kikai-Akahoya eruption on human society in Kyushu]. *Ritsumeikan Pan-Pac. Civ. Stud.* **4**, 21–31 (2020).

20. J. Uchiyama, M. Kuwahata, Y. Kowaki, N. Kamijō, J. Talipova, K. Gibbs, P. D. Jordan, S. Isaksson, Disaster, survival and recovery: The resettlement of Tanegashima Island following the Kikai-Akahoya “super-eruption”, 7.3ka cal BP. *Antiquity* **97**, 557–575 (2023).

21. J. Uchiyama, M. Kuwahata, F. Riede, P. D. Jordan, Exploring the environmental and cultural consequences of the 8.2 ka cooling event in Kyushu, Southwestern Japan. *Quat. Environ. Hum.* **3**, 100081 (2025).

22. J. Park, J. Park, S. Yi, J. C. Kim, E. Lee, Q. Jin, The 8.2 ka cooling event in coastal East Asia: High-resolution pollen evidence from southwestern Korea. *Sci. Rep.* **8**, 12423 (2018).

23. S. Uesawa, K. Toshida, S. Takeuchi, D. Miura, Creating a digital database of tephra fallout distribution and frequency in Japan. *J. Appl. Volcanol.* **11**, 14 (2022).

24. E. R. Crema, Statistical inference of prehistoric demography from frequency distributions of radiocarbon dates: A review and a guide for the perplexed. *J. Archaeol. Method Theory* **29**, 1387–1418 (2022).

25. A. Bevan, S. Colledge, D. Fuller, R. Fyfe, S. Shennan, C. Stevens, Holocene fluctuations in human population demonstrate repeated links to food production and climate. *Proc. Natl. Acad. Sci. U.S.A.* **114**, E10524–E10531 (2017).

26. M. A. Chaput, B. Kriesche, M. Betts, A. Martindale, R. Kulik, V. Schmidt, K. Gajewski, Spatiotemporal distribution of Holocene populations in North America. *Proc. Natl. Acad. Sci. U.S.A.* **112**, 12127–12132 (2015).

27. E. R. Crema, A. Bevan, S. Shennan, Spatio-temporal approaches to archaeological radiocarbon dates. *J. Archaeol. Sci.* **87**, 1–9 (2017).

28. E. R. Crema, S. Carrignon, S. Shoda, C. J. Stevens, Regional variations in the demographic response to the arrival of rice farming in prehistoric Japan. *Antiquity* **98**, 1290–1305 (2024).

29. P. Onkamo, J. Kammonen, P. Pesonen, T. Sundell, E. Moltchanova, M. Oinonen, M. Haimila, E. Arjas, Bayesian spatiotemporal analysis of radiocarbon dates from eastern Fennoscandia. *Radiocarbon* **54**, 649–659 (2012).

30. F. Riede, Doing palaeo-social volcanology: Developing a framework for systematically investigating the impacts of past volcanic eruptions on human societies using archaeological datasets. *Quat. Int.* **499**, 266–277 (2019).

31. J. R. M. Allen, B. Huntley, Effects of tephra falls on vegetation: A Late-Quaternary record from southern Italy. *J. Ecol.* **106**, 2456–2472 (2018).

32. S. Hotes, A. P. Grootjans, H. Takahashi, K. Ekschmitt, P. Poschlod, Resilience and alternative equilibria in a mire plant community after experimental disturbance by volcanic ash. *Oikos* **119**, 952–963 (2010).

33. P. D. M. Hughes, G. Mallon, A. Brown, H. J. Essex, J. D. Stanford, S. Hotes, The impact of high tephra loading on late-Holocene carbon accumulation and vegetation succession in peatland communities. *Quat. Sci. Rev.* **67**, 160–175 (2013).

34. H. Machida, F. Arai, Akahoya ash-A Holocene widespread tephra erupted from the Kikai caldera, South Kyushu, Japan. *Quat. Res.* **17**, 143–163 (1978).

35. S. Suto, T. Inomata, H. Sasaki, S. Mukoyama, Data base of the volcanic ash fall distribution map of Japan. *Bull. Geol. Surv. Japan* **58**, 261–321 (2007).

36. M. R. Auker, R. S. J. Sparks, L. Siebert, H. S. Crosweller, J. Ewert, A statistical analysis of the global historical volcanic fatalities record. *J. Appl. Volcanol.* **2**, 2 (2013).

37. C. S. Witham, Volcanic disasters and incidents: A new database. *J. Volcanol. Geotherm. Res.* **148**, 191–233 (2005).

38. M. Kuwahata, "Todoroki shiki doki [Todoroki type pottery]" in *Jomon Doki Soran [Handbook of Jomon Pottery]*, T. Kobayashi, Ed. (UM Promotion, 2008), pp. 328–335.

39. S. Shimomura, " Kyūshū shutsudo no ketsujō mimikazari ni kansuru kisoteki kentō [Fundamental study on slit-earrings (Ketsujō-mimikazari) excavated from Kyushu]" in *Senshigaku Kōkogaku Ronkyū [Studies in Prehistory and Archaeology (Collected Papers)] V* [Tatsuta Kōkokai (Tatsuta Archaeology Society), 2010], pp. 191–221.

40. Y. Ōtsubo, Ed. "Dai 3-shō: Kyūshū ni okeru ketsujō mimikazari no keifu [Chapter 3: The developmental sequence of slit-earrings in Kyushu]" in *Jōmon Tama Bunka No Kenkyū* (Yūzankaku, 2015), pp. 37–48.

41. H. Kawamichi, "Tsugume-no-hana iseki no kujira ryō [Whaling at the Tsugume-no-hana Site]" in *Jōmon Jidai No Kōkogaku [Jomon Archaeology] 5*, Y. Kosugi, Ed. (Dōseisha, 2007), pp. 163–169.

42. H. Kawamichi, Ishimori to kyoshi sentōki no nisha: Jōmon jidai sekisei gyorōgu no shosō [Harpoons and serrated points: Aspects of stone fishing implements in the Jōmon Period]. *Res. Bull. Nagasaki Prefect. Archaeol. Cent.* **3**, 1–10 (2013).

43. K. Mizunoe, *Kyushu Jomon Bunka No Kenkyu [Study of the Jomon Culture in Kyushu]* (Yuzankaku, 2012).

44. Kiyotake Town Board of Education, "Kiyotake Town Archaeological Report No. 22: Numerikawa No. 2 site" (Kiyotake Town Board of Education, 2007).

45. H. Dōgome, "Sobata shiki doki [Sobata type pottery]" in *Jomondoki Souran [Handbook of Jomon Pottery]*, T. Kobayashi, Ed. (UM Promotion, 2008), pp. 336–343.

46. M. Shimada, Kokuyōseki kara miru kōryū no ichiyōsō [An aspect of interaction seen through obsidian]. *Newslett. South. Kyushu Jōmon Res. Group* **12**, 59–66 (1998).

47. M. Akinari, "Funabiki chiku isekigun ni okeru Jomon jidai Sōki no sekki no yōsō [Lithic assemblages of the Initial Jomon period in the Funabiki Site Complex]" in *Heisei 26-Nendo Miyazaki Kōkogakkai Kenkyūkai Shiryōshū: Kaigarabun to Oshigatamon [Proceedings of the 2014 Miyazaki Archaeological Society Workshop: Kaigaramon (Shell-Impressed) and Oshigatamon (Impressed-Pattern) Pottery]* [Miyazaki Kōkogakkai Kennan Reikai Jikkō Iinkai (Organizing Committee of the Southern Miyazaki Regional Meeting, Miyazaki Archaeological Society), 2015], pp. 65–80.

48. Y. Furusawa, Genkai-nada tōshoiki o chūshin ni mita Jōomon jidai Nikkan doki bunka kōryū no seikaku: Yayoi jidai sōki to no hikaku [The nature of Jōmon pottery cultural interaction between Japan and Korea with a focus on the Genkai Sea island region: A comparison with the Early Yayoi period]. *Bull. Dept. Archaeol. Univ. Tokyo* **28**, 27–80 (2014).

49. F. Riede, U. Kierdorf, The eruption of the Laacher See volcano (∼13,000 years BP) and possible fluoride poisoning amongst contemporaneous wildlife and human foragers—Outline of a hypothesis and the way to test it. *Int. J. Osteoarchaeol.* **30**, 855–871 (2020).

50. F. Riede, O. Bazely, Testing the "Laacher See hypothesis": A health hazard perspective. *J. Archaeol. Sci.* **36**, 675–683 (2009).

51. F. Riede, J. M. Wheeler, Testing the "Laacher See hypothesis": Tephra as dental abrasive. *J. Archaeol. Sci.* **36**, 2384–2391 (2009).

52. F. Riede, Past-forwarding ancient calamities. Pathways for making archaeology relevant in disaster risk reduction research. *Humanities* **6**, 79 (2017).

53. Y. Kudo, M. Sakamoto, M. Hakozaki, C. J. Stevens, E. R. Crema, An archaeological radiocarbon database of japan. *J. Open Archaeol. Data* **11**, 11 (2023).

54. E. R. Crema, S. Shoda, A Bayesian approach for fitting and comparing demographic growth models of radiocarbon dates: A case study on the Jomon-Yayoi transition in Kyushu (Japan). *PLOS ONE* **16**, e0251695 (2021).

55. P. de Valpine, D. Turek, C. J. Paciorek, C. Anderson-Bergman, D. T. Lang, R. Bodik, Programming with models: Writing statistical algorithms for general model structures with NIMBLE. *J. Comput. Graph. Stat.* **26**, 403–413 (2017).

56. P. de Valpine, C. Adler, D. Turek, N. Michaud, C. Anderson-Bergman, F. Obermeyer, C. Wehrhahn Cortes, A. Rodrìguez, D. Temple Lang, S. Paganin, NIMBLE: MCMC, Particle Filtering, and Programmable Hierarchical Modeling, Zenodo (2020); https://doi.org/10.5281/zenodo.1211190.

57. E. R. Crema, nimbleCarbon: Models and Utility Functions for Bayesian Analyses of Radiocarbon Dates with NIMBLE, GitHub (2022); https://github.com/ercrema/nimbleCarbon.

58. A. Gelman, D. B. Rubin, Inference from iterative simulation using multiple sequences. *Stat. Sci.* **7**, 457–472 (1992).

59. M. Ester, H.-P. Kriegel, J. Sander, X. Xu, “A density-based algorithm for discovering clusters in large spatial databases with noise,” in *Proceedings of the Second International Conference on Knowledge Discovery and Data Mining* (AAAI Press, 1996), pp. 226–231.

60. E. R. Crema, A. Bevan, Inference from large sets of radiocarbon dates: Software and methods. *Radiocarbon* **63**, 23–39 (2021).

61. E. Pebesma, R. Bivand, *Spatial Data Science: With Applications in R* (Chapman and Hall/CRC, 2023).

62. E. Pebesma, Simple features for R: Standardized support for spatial vector data. *R J.* **10**, 439 (2018).

63. P. J. Reimer, W. E. N. Austin, E. Bard, A. Bayliss, P. G. Blackwell, C. B. Ramsey, M. Butzin, H. Cheng, R. L. Edwards, M. Friedrich, P. M. Grootes, T. P. Guilderson, I. Hajdas, T. J.

Heaton, A. G. Hogg, K. A. Hughen, B. Kromer, S. W. Manning, R. Muscheler, J. G. Palmer, C. Pearson, J. van der Plicht, R. W. Reimer, D. A. Richards, E. M. Scott, J. R. Southon, C. S. M. Turney, L. Wacker, F. Adolphi, U. Büntgen, M. Capano, S. M. Fahrni, A. Fogtmann-Schulz, R. Friedrich, P. Köhler, S. Kudsk, F. Miyake, J. Olsen, F. Reinig, M. Sakamoto, A. Sookdeo, S. Talamo, The IntCal20 Northern Hemisphere radiocarbon age calibration curve (0–55 cal kBP). *Radiocarbon* **62**, 725–757 (2020).

64. M. Plummer, N. Best, K. Cowles, K. Vines, CODA: Convergence diagnosis and output analysis for MCMC. *R News* **6**, 7–11 (2006).
